# Biosecurity in the age of synthetic nucleic acids: modernizing the law to manage emerging threats

**DOI:** 10.1093/jlb/lsag005

**Published:** 2026-04-28

**Authors:** Ayelet Berman

**Affiliations:** Saw Swee Hock School of Public Health, Asia Centre for Health Security, National University of Singapore

**Keywords:** synthetic nucleic acids, biosecurity, synthetic nucleic acid/ gene synthesis screening, transnational new governance, Biological Weapons Convention, sequences of concern

## Abstract

The rapid expansion of synthetic biology has transformed research and innovation but has also created profound biosecurity challenges. Synthetic nucleic acid (SNA) technologies, which allow genetic material to be synthetically created, enable scientific progress but also lower the barriers to constructing or enhancing dangerous pathogens. This article argues that the governance of SNA should be grounded in a transnational new governance approach that combines binding international obligations with harmonized technical standards. It assesses the fitness of current regimes—the International Health Regulations, the Biological Weapons Convention (BWC), UN Security Council Resolution (UNSCR) 1540, and national biosecurity laws—and finds that while these instruments already impose binding obligations to prevent misuse of biological agents, their terms remain outdated and their application fragmented. Most states lack explicit SNA order screening requirements, and voluntary private standards such as those of the International Gene Synthesis Consortium and ISO 20688-2 remain inadequate for managing this global risk. The article recommends modernizing international law by clarifying that existing treaties cover synthetic biology, developing harmonized global screening standards, and updating national legislation to mandate and incentivize SNA order screening. It further proposes leveraging market access and funding power to drive global practice. Ultimately, safeguarding innovation in the age of SNA requires aligning law to manage the risks of emerging biotechnologies.

## I. INTRODUCTION

The accelerating accessibility of synthetic nucleic acid (SNA) technologies marks a new frontier in biotechnology—and a new era of biosecurity risk. While synthetic biology fuels innovation across medicine, agriculture, and energy, it has also lowered the barriers to constructing or modifying pathogens, including those with pandemic potential.^1^ Combined with artificial intelligence (AI), a globalized bioeconomy,^2^ and benchtop synthesis devices, SNA technologies enable capabilities once confined to advanced laboratories to be accessible worldwide.

Yet the legal frameworks that govern biosecurity—nationally and internationally—remain anchored in an earlier era. They were designed to control pathogens of natural origin, not synthetic constructs created from digital code. The core international legal obligations—the Biological Weapons Convention (BWC), UN Security Council Resolution 1540, and the International Health Regulations (IHR)—were negotiated decades before the advent of modern synthetic biology. Nationally, most legal systems lack comprehensive biosecurity regulation. Those that do regulate the handling of dangerous pathogens typically rely on select agent laws, export controls, or anti-terrorism measures, yet few extend these frameworks to SNAs. As a result, the global regulatory landscape is outdated and fragmented, leaving major gaps in oversight.

In this governance vacuum, the main standards governing SNA screening have emerged not from governments or international organizations, but from private industry. The International Gene Synthesis Consortium (IGSC) and the International Organization for Standardization (ISO 20688-2) have developed voluntary screening protocols that require providers to screen orders. While these standards are followed by some providers, they are not legally binding, and many companies operate outside these frameworks altogether. The result is a global biosecurity system largely governed by private standards that lack legal force and are limited in scope—a situation untenable for technologies with potentially catastrophic consequences.

Against this background, the article first seeks to identify what an effective regulatory approach for SNA should look like. Building on a transnational new governance approach, it argues that a binding international agreement is essential because of the nature of SNA itself. Their production and use are inherently global, as digital sequences and synthesized fragments move easily across borders; the system is only as strong as its weakest link, since unregulated jurisdictions can become safe havens; and the technology is technically complex and fast-evolving in an era of AI, requiring expertise in the development of regulation. National or private measures alone cannot address these challenges. An international agreement mandating sequence and customer screening, supported by harmonized international standards, would ensure consistency across jurisdictions and prevent regulatory arbitrage.

Consequently, the article examines existing legal frameworks to assess whether they are fit for purpose—asking whether new laws are required or whether existing instruments can be adapted to govern SNA effectively. It makes the following main arguments and findings.

The article argues that existing international instruments can and should be interpreted and implemented in a technology-neutral manner. The BWC, UNSCR 1540, IHR, and the new pandemic agreement already impose obligations on states to prevent the misuse and proliferation of biological agents, regardless of their origin or method of production. States, therefore, have a legal duty to adopt domestic measures that mitigate the risks of SNAs, even if not expressly mentioned in these treaties. Clarifying this interpretation—through statements at BWC Review Conferences or WHO forums—would help modernize these frameworks without reopening negotiations.

Recognizing that international agreement may be difficult in the current geopolitical climate, coalitions of willing states and major economies can use their market power to drive global practice. The USA, the European Union, China, and other countries home to large bioeconomies could condition market access, procurement contracts, and public funding on compliance with screening requirements, creating powerful incentives for providers worldwide. Such ‘club’ standards, when adopted by influential states, often evolve into *de facto* global norms, as demonstrated in other transnational regulatory domains from pharmaceuticals to banking supervision.

Further, national legislation must be developed and updated to close critical gaps. Most countries lack any biosecurity framework, and where one exists, it rarely addresses SNAs. Even in advanced jurisdictions, laws often focus on natural pathogens, leaving synthetic constructs outside their scope. Only a few countries—most notably the USA and the United Kingdom—have modernized their frameworks and issued governmental guidance on screening.

Finally, the article shows that while private governance fills a temporary gap, it cannot serve as the cornerstone of global biosecurity. Voluntary screening standards—such as those of IGSC and ISO—are helpful but suffer from predictable shortcomings: uneven adoption and no enforcement. In a field where misuse could lead to catastrophic outcomes, relying on self-regulation is too risky.

It is important to highlight that the article focuses on the regulation of access to synthesized nucleic acids, in other words, the point at which digital sequences are translated into physical, biological materials. It does not cover the regulation of AI-bio models. While the AI-bio interface raises critical and increasingly urgent governance questions, it involves distinct regulatory challenges and tools that go beyond the scope of this article.

In sum, this article makes several central claims and findings. First, SNAs pose a transnational, potentially catastrophic risk that cannot be effectively governed through national or private measures alone; they require an international and multi-level legal response. Second, the foundations for such a regime already exist in international law, which should be interpreted and modernized to encompass synthetic biology. Third, national biosecurity frameworks must be developed where they do not exist and updated where foundations already exist. Fourth, achieving global health security requires an inclusive, transnational, and multi-level governance model that combines hard law (binding treaties and national regulation), soft law (technical guidance and standards), and hybrid mechanisms (public–private and multi-stakeholder cooperation).

The article is organized as follows: Section II sets out the benefits and risks of SNA. Section III lays out the provider-screening approach. Section IV develops the transnational new governance framework. Section V assesses whether existing legal frameworks are fit for purpose. Section V.A evaluates international governance. Section V.B examines national governance. Section V.C reviews private governance. Section VI concludes and sets out recommendations for modernizing biosecurity regulation.

## II. THE BENEFITS AND RISKS OF SNA

Nucleic acid synthesis is a technology that enables the artificial creation of DNA or RNA strands directly from digital genetic sequences. In the past, genetic material had to be physically extracted from natural organisms, making the process time-consuming, costly, and reliant on limited biological samples. However, with recent advances in synthetic biology, a DNA or RNA sequence can now be digitally retrieved from a database, synthesized into nucleotide strands, and subsequently assembled into functional genes or even entire genomes. This shift has transformed how biological research and development are conducted, with nucleic acid synthesis becoming a widely accessible and increasingly affordable technology.^3^

### I‌I.A. The Benefits of SNA

The applications of this technology span multiple sectors, including medicine, agriculture, environmental science, vaccine development, and industrial manufacturing. For example, synthetic yeast is used in the production of food ingredients,^4^ while synthetic insulin reduces the costs of managing diabetes,^5^ and synthetic microorganisms are being developed to produce biofuels and biodegradable materials.^6^ Against this background, many countries are adopting bioeconomy strategies to advance their economies through synthetic biology and other biotechnologies.^7^ This has led to a booming bioeconomy, with gene synthesis revenue expected to reach around $10.6 billion by 2030.^8^ According to some reports, the five countries generating the greatest demand for the global gene synthesis market are the USA, China, Japan, the United Kingdom, and South Korea.^9^

### I‌I.B. The Risk of Misuse

Despite its immense promise, nucleic acid synthesis presents new and more challenging biosecurity risks.^10^ Biosecurity refers to ‘unauthorized access, loss, theft, misuse, diversion, or release of biological agents’.^11^ This technology allows the creation of dangerous pathogens from scratch. Scientists have already demonstrated the feasibility of synthesizing dangerous viruses such as polio, the 1918 influenza virus, and horsepox.^12^ Using publicly available sequence data, it is now possible to synthesize the full genome of a pathogen, assemble it, and—under the right laboratory conditions—reboot the organism.^13^ In other cases, synthetic tools could be used to modify non-pathogenic organisms in ways that enhance transmissibility or virulence^14^ by introducing traits that create resistance to treatments or vaccines. For example, a harmless strain of *Escherichia coli* could be engineered to produce toxins. While in the past there was some skepticism about the magnitude of the risk,^15^ recent technological advances and commercial factors^16^ have significantly increased the risk of this technology and made it hard to dismiss,^17^ as follows:

- **AI-enabled biological design tools** ^18^ can be exploited to engineer novel pathogens or enhance the virulence of existing ones. In the absence of guardrails on AI development and use, this risk is particularly acute.^19^
- **AI chatbots** may lower barriers to entry by providing malicious actors with technical guidance for developing biological weapons.^20^
- **Open-access databases** such as GenBank and GISAID, while designed to advance collaboration, also expose vast amounts of pathogen sequence data to potential misuse.^21^
- **Declining synthesis costs** enable a growing number of laboratories, startups, and providers worldwide to access nucleic acid synthesis technologies.^22^
- **Benchtop nucleic acid synthesizers**, increasingly manufactured and sold, extend powerful synthesis capabilities to individuals outside traditional laboratory settings, often without adequate oversight.^23^

Nucleic acid synthesis thus represents both a powerful driver of innovation and a potential source of catastrophic harm. The following sections examine how these concerns can be managed through screening practices and regulatory frameworks.

## III. SCREENING OF ORDERS BY PROVIDERS: THE CORNERSTONE OF BIOSECURITY OVERSIGHT

Despite biosecurity risks, access to SNAs has often proven alarmingly easy. In 2006, a journalist from *The Guardian* successfully ordered a fragment of the smallpox virus DNA to a private address.^24^ More recently, in 2024, MIT researchers demonstrated how easily gene fragments of the 1918 pandemic influenza virus could be ordered.^25^

These incidents highlighted the minimal barriers to acquiring hazardous genetic material and spurred calls for provider-based screening of orders. Because providers and benchtop manufacturers act as gatekeepers—receiving and deciding on orders—they are best placed to block access by malicious actors. Accordingly, there is a broad consensus that provider-based order screening is a key safeguard for preventing misuse.^26^ The following section outlines the two main conceptual pillars—sequence screening and customer legitimacy screening—and the technical challenges each presents, as well as the regulation of users. Concrete examples of this approach include US and UK guidance, the IGSC harmonized protocol, and the ISO standard, as detailed below in Sections V.B and V.C.

### I‌II.A. Sequence Screening

When a provider receives an order, the ordered sequence needs to be screened against a *list* of regulated pathogens. Listed pathogens are biological agents and toxins that appear on formal international or national control lists because of their potential to cause serious harm. Yet advances in AI and biotechnology are increasingly challenging the effectiveness of this traditional list-based approach, as follows.

#### I‌II.A.1. Sequences of Concern

The convergence of AI and synthetic biology now enables the design of sequences that, while absent from existing regulatory lists, may nonetheless be dangerous. These so-called ‘sequences of concern’ (SOCs) do not appear on current lists of regulated pathogens, yet they may encode functions that increase virulence, transmissibility, or resistance to treatment.^27^ Broadly understood, SOCs encompass any sequences that could be misused to cause significant harm, not only those currently listed.^28^ This reality poses a serious challenge for screening. A related challenge is that increasingly shorter nucleic acid fragments—some harmless in isolation—can be connected to create a dangerous pathogen.^29^ Thus, smaller DNA or RNA fragments that are not on control lists could bypass screening if ordered separately—and later be assembled into a dangerous sequence.

Against this background, experts have urged moving beyond list-based screening toward functional approaches that can detect genetic elements associated with dangerous biological functions. The technical details of this SOC model are still evolving, with groups such as the International Biosecurity and Biosafety Initiative for Science (IBBIS), Engineering Biology Research Consortium,^30^ or Microsoft researchers,^31^ developing methods. Proposed features include retaining best-match and taxonomy checks while adding function-aware modules.^32^ Because no central databases of SOCs currently exist, some have suggested creating secure repositories—though their development presents significant technical challenges.^33^ For instance, Baker and Church propose storing all synthetic gene sequences and AI-generated synthesis data in repositories accessible only during emergencies.^34^ Another unresolved issue is the choice of screening parameters: tighter thresholds (eg, flagging any 50-bp match) may capture more risks but also increase false positives.^35^ In sum, SOC screening remains under development, and its methods continue to be refined.^36^ The technical modalities of screening fall beyond the scope of this article.

#### I‌II.A.2. Benchtop Devices

A further complicating factor is that previously, nucleic acid sequences had to be ordered from providers, who could, at least in principle, be made responsible for screening. Now, benchtop devices allow users to synthesize fragments in-house. This decentralization will make it much harder to monitor who is using the technology and for what purpose, effectively bypassing centralized screening systems. As benchtop synthesizers become more widely adopted, there is discussion regarding the need to incorporate screening capabilities into these devices or cloud-based platforms.^37^

### I‌II.B. Customer Screening

If a sequence raises a red flag, the provider (or other actors in the distribution chain) must verify the legitimacy of the customer. This is a complex task. While some customers may appear on international or national watchlists (e.g, lists of sanctioned individuals or suspected terrorists), these lists vary across jurisdictions. Additionally, a malicious actor may operate within a legitimate institution or pose as a qualified researcher, making detection difficult.^38^ To address these risks, a customer due diligence or ‘know your customer’ procedure, such as those commonly applied in financial compliance regimes, could arguably be followed.^39^ Another option is a licensing regime under which only customers with government-issued licenses may procure SNA, but such an approach would severely restrict the market. The details of such regimes are beyond the scope of this article.

### I‌II.C. Incentivizing Providers through the Regulation of Users

To further strengthen compliance with screening practices, there is also a growing recognition that regulating end users can incentivize screening practices. Users—whether academic researchers, commercial labs, or other actors—can significantly influence the system by ordering sequences only from providers who screen (Note: In this article, the term ‘users’ is used broadly and may refer to end users, principal users, or customers). Governments can support this through legal requirements or by linking funding to compliance, while users themselves can reinforce this practice through self-regulation and professional norms. Examples of this regulatory approach are laid out below (Section V), and can be found in Executive Order 14292,^40^ the WHO Global Guidance Framework for the Responsible Use of the Life Sciences: Mitigating Biorisks and Governing Dual-Use Research,^41^ the *Guiding Principles on Responsible Use of AI for Protein Design*.^42^ and International Genetically Engineered Machine’s (iGEM) guidelines on responsible synthetic biology.^43^

**Diagram 1:**
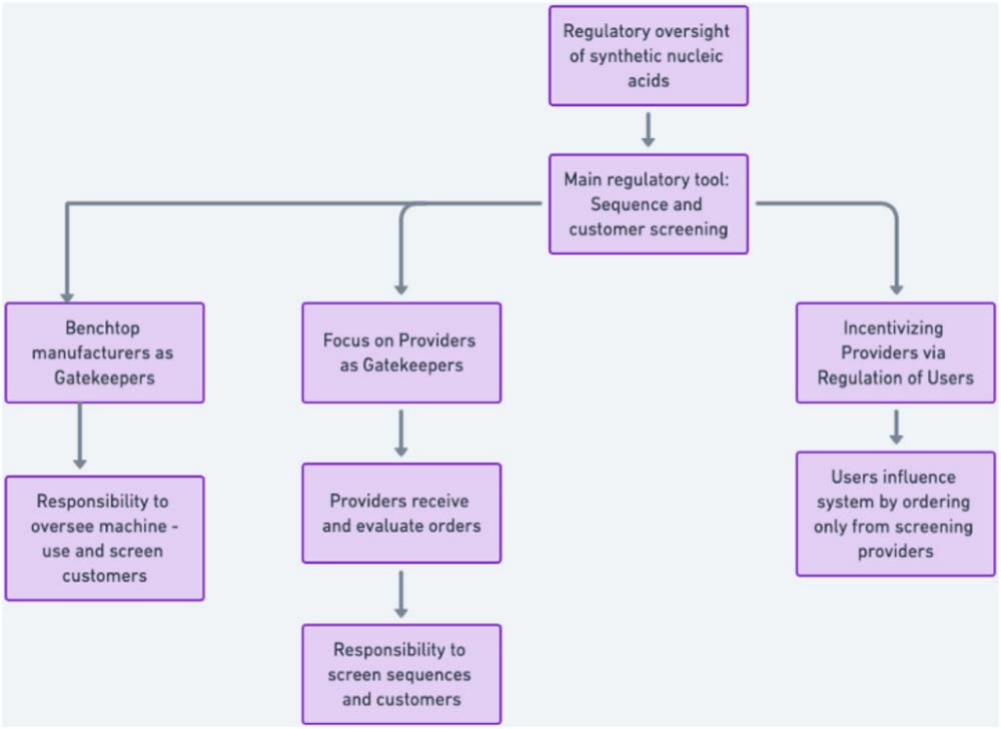
Screening: providers, benchtop manufacturers and users.

**Diagram 2:**
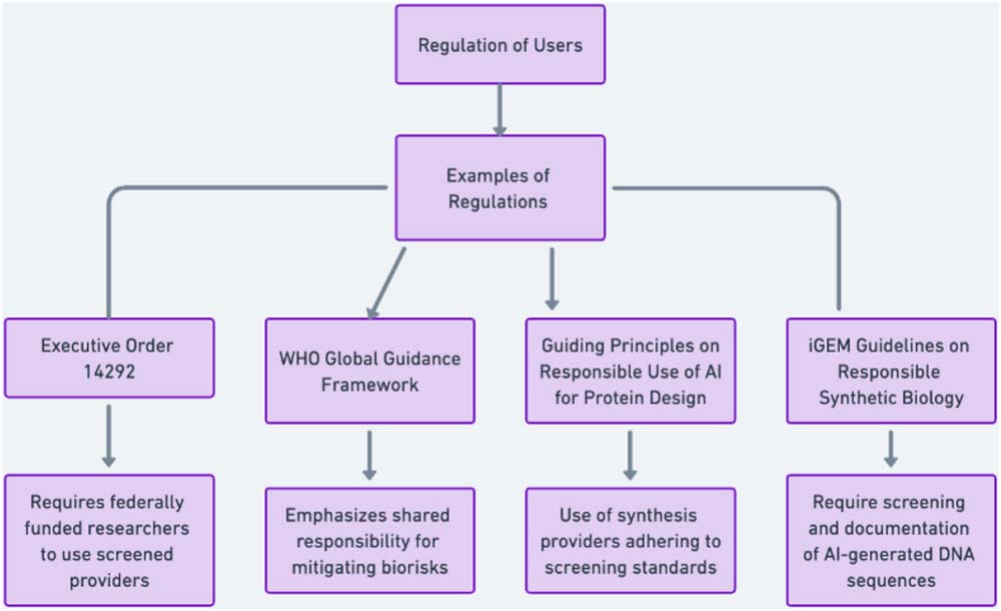
Regulation of users.

In sum, provider-based screening has become the preferred approach for mitigating the risks of SNAs. The structural components of the screening model are depicted in Diagram 1, and examples of regulations governing suers are depicted in Diagram 2. In the absence of regulation governing the use of AI in life-sciences development, the need to safeguard outputs becomes even more critical. Yet significant technical hurdles remain. Addressing these challenges will require ongoing scientific and technical innovation – issues that are beyond the scope of this article.

The next section therefore turns to a different question: how can screening be most effectively integrated into regulatory frameworks?

## IV. GOVERNANCE APPROACHES

Before examining existing legal and regulatory frameworks, it is useful to step back and ask a higher-level question: what kind of governance model is best suited to managing the biosecurity risks of SNAs?

Regulation of SNA is especially complex for three reasons:

- **Global**: SNA presents an inherently global challenge. Orders can be placed across borders, and any resulting outbreak could spread internationally, with potentially catastrophic consequences.
- **Weakest link**: It suffers from the weakest-link problem: the security of all depends on the least regulated jurisdiction.^44^ These two factors make international cooperation indispensable.
- **Technical**: The risks are highly technical and rapidly evolving. As outlined above, advances in synthetic biology and AI are generating new challenges that demand cutting-edge expertise and rapid adaptation. Because governments often lack the capacity to keep pace with these developments, effective governance depends on drawing on the knowledge and expertise of industry and the scientific community. At the same time, involving civil society is essential to ensure that public interests in safety and security are represented.

In this context, the transnational new governance approach offers a useful lens.^45^ Unlike traditional regulatory models that rely exclusively on binding, hierarchical rules (‘hard law’),^46^ it recognizes the value of a spectrum of instruments. At one end is hard law: treaties and mandatory national laws that are legally binding and enforceable.^47^ At the other end is soft law: non-binding instruments such as voluntary standards, guidelines, or principles that can be adopted quickly and flexibly, though without the force of law. Between these poles are hybrid models such as public-private regulation, which blend public authority with private expertise. Further, authority is not held by governments alone but is distributed across networks that include states, international organizations, industry associations, and civil society. The premise is that in a globalized world marked by interdependence and rapid technological change, effective governance must be multi-level (international, national), multi-actor (involving states, international organizations, industry associations, and civil society), and multi-instrument (using the full spectrum from hard to soft law).^48^

**Diagram 3:**
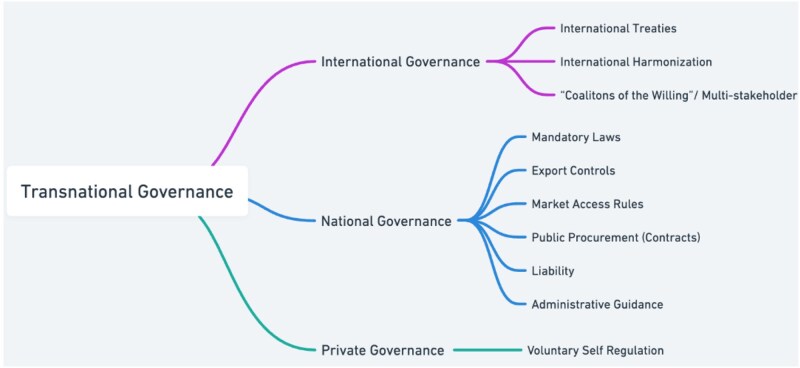
Transnational governance approaches.

Against this backdrop, seeking to identify an effective model for regulating SNAs, the next section examines models for regulating SNA across international, national, and private governance. Each level employs hard law, hybrid, and soft law tools, and each presents both advantages and limitations for regulating SNA. Diagram 3 depicts the transnational governance approach laid out below.

### IV.A. International Governance

#### IV.A.1. International Treaties (Hard Law)

Legally binding, multilateral, international agreements, also referred to as treaties, are the strongest governance tool. They create clear obligations for all states, reduce fragmentation, and establish a level playing field for industry and states by ensuring that all providers comply with the same rules. Global biosecurity is only as strong as its weakest link, and unregulated providers anywhere can become safe havens—undermining health security worldwide. Thus, given the global nature of the SNA market, multilateralism is the ideal approach, as it would require all states to implement screening obligations.

In principle—as we shall see below—there is no need to negotiate a new treaty. Existing instruments—the BWC and UN Security Council Resolution 1540—already provide a legal foundation, requiring only clarification that they require SNA screening. Yet in today’s geopolitical climate, achieving consensus on new or expanded obligations is likely to be difficult in practice.

#### IV.A.2. International Harmonization (Soft Law)

International harmonization refers to the alignment of technical standards across countries. It is best understood as a complement to binding treaties rather than a substitute: while treaties can establish the obligation to screen, harmonized standards set out the technical requirements for how screening should be conducted in practice. By reducing regulatory fragmentation, harmonization promotes consistent implementation of standards across jurisdictions. Precedents exist in many other transnational domains, such as Codex Alimentarius for food safety and International Civil Aviation Organization (ICAO) for aviation.^49^

For SNA, no such standards yet exist, but the BWC or World Health Organization (WHO) could serve as natural fora for their development. Including industry and scientific experts would be essential,^50^ as they hold the technical knowledge needed. However, even technical harmonization has become difficult in the current polarized environment.^51^

#### IV.A.3. International Multi-Stakeholder Cooperation (Hybrid)

In light of the potential obstacles to binding treaties and international harmonization, multi-stakeholder cooperation at the international level may offer a practical path forward. In this model, willing governments, industry, and civil society collaborate to develop voluntary guidance or standards that can then be adopted into national frameworks. Well-established precedents exist in other fields, such as the Basel Committee on Banking Supervision global banking standards, International Conference on Harmonization (ICH) pharmaceutcial guidelines, International Cooperation for Assigned Names and Numbers (ICANN) standards, or International Accounting Standards Board (IASB) standards.^52^ Through their combined market power, these ‘clubs’ have had a global impact way beyond their membership.

This model offers four main advantages. First, it reflects the political feasibility of ‘coalitions of the willing’:^53^ groups of states with developed or emerging bioeconomies can move forward even when universal consensus is blocked. Second, it leverages the technical expertise of industry, ensuring that screening protocols are both scientifically robust and practically implementable.^54^ Third, it incorporates the participation of civil society and scientific actors, which helps safeguard the public interest in safety and reduces the risk of regulatory capture to industry interests.^55^ Fourth, it draws on the market leverage of participating states: their combined market power will make compliance necessary for companies seeking to compete, thereby extending the influence of the coalition even beyond its membership.

Although such ‘club’ collaboration has not yet emerged in the context of SNA, it could prove highly beneficial.^56^ If the guidelines developed by such a body were subsequently integrated into national frameworks, they could generate meaningful global change despite the absence of universal consensus.

#### IV.A.4. Conclusion: Promise and Limit of International Governance

In sum, international governance is the most appropriate level for addressing SNA risks, given their transnational nature and weakest link problem. Binding agreements and harmonized standards would offer the greatest protection, and industry and civil society involvement is crucial to ensure technical feasibility and proper safety guardrails. Yet collective action traps make such solutions difficult to achieve.

In this environment, coalitions of the willing or multi-stakeholder initiatives represent a pragmatic alternative. By pooling their authority and market power, such clubs can set *de facto* global standards: their combined influence makes compliance necessary for companies seeking to compete, extending their impact well beyond formal membership. Over time, these initiatives can generate momentum, and serve as a stepping stone for broader international agreement. For an overview of international governance approaches, see Table 1.

**Table 1:** International governance approaches for regulating SNAs

Type of governance	Main characteristics	Advantages for regulating SNA	Disadvantages/limitations
**1. International treaties (hard law)**	Legally binding agreements among states; create enforceable obligations; strongest form of international governance.	• Establish clear, uniform obligations across countries. • Reduce fragmentation and ensure a level playing field for states and industry. • Provide legal certainty and accountability. • Existing instruments (BWC, UNSCR 1540) already offer a foundation.	• Negotiating or expanding treaties is politically difficult in today’s geopolitical climate. • Time-consuming to achieve universal consensus. • Compliance and enforcement can still vary across states.
**2. International harmonization (soft law)**	Alignment of technical standards and procedures across jurisdictions, typically voluntary and complementary to treaties.	• Promotes consistency and interoperability of screening practices. • Encourages technical precision and shared benchmarks. • Builds on successful models (e.g. Codex, ICAO). • Can complement existing legal obligations.	• Lacks binding force—depends on voluntary adoption. • Technically demanding and politically challenging in polarized environments. • Risk of uneven implementation.
**3. International multi-stakeholder cooperation (hybrid)**	Collaborative ‘club’ model involving willing governments, industry, and civil society to develop voluntary global standards or guidance.	• Politically feasible alternative when treaties stall. •Leverages technical expertise of industry and scientists. •Incorporates civil society, reducing risk of regulatory capture. • Uses market power of participants to drive global compliance beyond membership. • Proven effectiveness in other sectors (e.g. Basel Committee on Banking Supervision, Iernational Conference on Harmonization (ICH, ICANN)).	• Voluntary nature limits coverage and enforcement. • Risk of fragmentation if multiple initiatives emerge. • Dependent on continued political and market momentum. • May lack legitimacy in eyes of non-participants.

**Table 2:** National governance approaches for regulating SNAs.

Type of governance	Main characteristics	Advantages for regulating SNA	Disadvantages/limitations
**1. Mandatory national regulation (hard law)**	Legally binding rules requiring providers to screen orders; creates enforceable domestic obligations.	• Establishes clear, uniform national standards. • Reduces reputational and competitive uncertainty among providers. • Provides a legal basis for requesting customer information. • Promotes accountability and compliance.	• Risk of overregulation may hinder innovation and competitiveness. • Costs fall disproportionately on smaller firms. • Without international alignment, may disadvantage domestic providers and shift orders abroad. • Politically difficult in absence of global consensus.
**2. Export controls (hard law)**	Restrictions on cross-border transfer of strategic goods, data, and technologies; implemented through licensing systems.	• Existing, well-understood mechanism under national security law. • Aligns with obligations under BWC and UNSCR 1540. • Reduces cross-border transfer of risky sequences. • Can be extended to include SNA screening.	• Covers only exports, not domestic transfers. • Creates uneven global playing field if adopted unilaterally.
**3. Market access rules (hard law)**	Conditioning entry into domestic markets on compliance with screening requirements.	• Powerful incentive when adopted by large markets (eg, EU, US, China). • Can create de facto global standards (as seen with EU General Data Protection Regulation (GDPR). • Encourages industry-wide adoption without global treaty.	• Requires large or coordinated markets to be effective. • High regulatory and enforcement capacity needed. • Limited influence for smaller or fragmented markets.
**4. Public procurement contracts (hard law)**	Embedding screening obligations into government contracts or funding agreements.	• Leverages government purchasing power to advance biosecurity. • Encourages compliance among providers serving publicly funded research. • Creates indirect but strong incentives for screening.	• Limited reach in economies with small public research sectors. • Does not affect private-sector providers. • Impact proportional to government demand share.
**5. Liability (hard law)**	Legal responsibility for harm arising from failure to comply with laws or reasonable precautions; enforced through administrative, tort, contract, or criminal law.	• Creates deterrence and promotes responsible practices. • Encourages adherence to screening guidelines as evidence of due care. • Provides recourse for victims of negligence.	• Difficult to prove causation or trace DNA origins. • Reactive rather than preventive—applies only after harm occurs. • Limited deterrence in low-enforcement settings.
**6. Administrative guidance (soft law)**	Non-binding policy documents issued by agencies to clarify expectations or procedures.	• Flexible and fast to issue and update. • Suitable for evolving technical fields such as synthetic biology and AI. • Allows rapid adaptation to new risks or technologies.	• Non-binding—compliance may be uneven. • Lacks enforcement mechanisms. • Effectiveness depends on linkage to binding law and liability protections.
**7. Licensing and certification (compliance tools)**	Mechanisms ensuring compliance by requiring authorization (licensing) or independent verification (certification).	• Ensures oversight and accountability. • Can restrict SNA use and sale to approved entities. • Certification can leverage international or private models (eg, FForest Stewardship Council (FSC), PIC/S). • Enhances trust and traceability.	• Adds administrative burden. • Requires institutional capacity for monitoring and audits. • Not effective as a stand-alone measure—must complement regulation.

### IV.B. National Governance

National governance plays two critical roles in governing SNAs. First, if an international agreement on screening were ever adopted, it would need to be implemented through domestic legislation. Second—and more importantly in the current absence of global consensus—national law provides the only binding regulatory tools available today. States therefore face the question of whether, and how, to act unilaterally despite the risks of competitive disadvantage.

A variety of governance mechanisms can be deployed at the national level, each with distinct advantages and drawbacks: mandatory regulation, export controls, market access, procurement contracts, guidance, and liability. These are further supported by compliance and enforcement measures such as licensing and certification.

#### IV.B.1. Mandatory National Regulation (Hard Law)

In the absence of an international agreement, some have called for mandatory national laws requiring providers to screen orders. Mandatory regulation would establish enforceable standards. The US National Science Advisory Board for Biosecurity (NSABB) has long supported this approach,^57^ and some companies also favor it, since binding rules create a level playing field,^58^ reduce reputational risks, and give providers a legal basis for requesting customer disclosures.^59^

In practice, however, without an international agreement that establishes a level playing field, states face a major dilemma: under-regulation, which risks catastrophic outcomes, versus overregulation, which could stifle innovation and competitiveness.^60^ Thus, as outlined below (Section V), there is currently no mandatory legal requirement for screening.

Concerns about overregulation are shaped by both national and international political economy considerations, as follows.

##### IV.B.1.i. Economic Considerations

- ***Costs and innovation***: Screening entails fixed investments in software and expertise. As the price of synthesis continues to fall, the relative burden of screening rises, particularly for smaller firms.^61^ Although initiatives such as IBBIS’s open-source Common Mechanism aim to reduce costs, concerns remain that regulation could dampen competition and slow innovation.^62^
- ***International competition***: Countries acting alone risk disadvantaging their providers vis-à-vis competitors in non-regulating jurisdictions.^63^ This creates incentives to shift orders abroad and makes smaller firms especially vulnerable to cost differentials.^64^

##### IV.B.1.ii. Developed and Emerging Bioeconomies vs. Developing Countries

Thus, without international alignment, most countries will hesitate to adopt binding rules for providers and/or for users. Yet mandatory screening is particularly important in countries with established or emerging bioeconomies, where the risks of misuse and harm—domestically and through potential spillover effects across borders—are higher. In these countries, governments should adopt screening requirements regardless of whether other countries do so. This obligation is reinforced by international law on transboundary harm, which requires states to take appropriate measures to prevent, or at least minimize the risk of, significant transboundary harm arising from activities within their jurisdiction or control.^65^ For countries that already have biosecurity frameworks in place, it would often require only targeted amendments to existing laws rather than entirely new legislation (I address that below, Section V).

As regards developing countries without bioeconomies, these countries may see little reason to adopt mandatory screening rules—unless required by an international agreement. However, without regulation, these countries could become safe havens or sites of misuse. From a global biosecurity perspective, this makes unregulated environments a serious risk and underscores the importance of securing international agreement. From a national perspective—beyond the potential desire of being a responsible global actor—there could also be a long-term interest in establishing sound regulatory frameworks that will help ensure growth of their bioeconomies in the future.

#### IV.B.2. Export Controls (Hard law)

Export controls protect national security by restricting the cross-border transfer of strategic goods, data, and technologies. By conditioning transfer on export licenses or prohibiting exports, they are designed to prevent dual-use items from reaching actors or countries of concern.^66^ Dual-use items are goods that have legitimate civilian applications but could also be misused for malicious purposes. Certain biological agents are considered dual-use goods as they can be used for beneficial purposes, such as the development of new vaccines, but they could also be used to develop biological weapons. Therefore, export control laws list biological agents that could be used in the development of biological weapons.

The advantage of relying on export controls to incentivize screening is that they are an established legal tool already embedded in many national systems. They provide a clear mechanism for overseeing international transfers and align with states’ obligations under the BWC and UN Security Council Resolution 1540 (see below, Section V.A). By targeting cross-border movements, they directly reduce the risk of dangerous sequences being sent to hostile actors abroad. Explicitly clarifying that export controls include screening requirements could therefore be an effective way to strengthen biosecurity.

That said, relying on export controls alone is insufficient. If only some states impose strict requirements, non-participating jurisdictions may gain a commercial advantage. This creates incentives for orders to be diverted to less-regulated markets. Further, they also do not apply to domestic transfers. Also, currently, most export controls apply only to listed pathogens and their genetic elements, leaving gaps for unlisted SOCs. To remain effective, control lists would need to be amended to capture these technical challenges.

In sum, export controls can serve as an important component of reducing SNA related risk, particularly when aligned with multilateral arrangements such as the Australia Group (AG) (see below, Section V). Yet on their own they are insufficient: they cannot prevent domestic misuse, and without a baseline international agreement, uneven adoption risks creating safe havens. They are best viewed as a necessary but partial tool that must be complemented by broader national regulation and, ideally, global coordination.

#### IV.B.3. Market Access Rules (Hard Law)

Market access rules make entry into a domestic market contingent on meeting specified safety and security requirements. In practice, this means that companies must demonstrate compliance—such as implementing screening practices or obtaining certification—before their products or services can be sold.^67^

When adopted by large and commercially attractive markets, this approach can be a powerful driver of global change. To secure access to these markets, providers would be strongly incentivized to adopt screening practices. The economic value of market access would outweigh the costs of screening, thereby creating powerful commercial incentives even in jurisdictions lacking binding screening rules. Over time, requirements of those markets often become *de facto* global standards. The impact of the EU’s General Data Protection Regulation (GDPR), which reshaped data practices worldwide, illustrates the extraterritorial influence of such measures.

But the effectiveness of market access rules to effectuate global change is heavily dependent on market size. Smaller or more fragmented markets lack the leverage to shape global behavior. In addition, designing and enforcing market access conditions requires significant regulatory capacity.

Market access rules thus represent a particularly promising tool for jurisdictions like the EU, the USA, or China, whose markets are large enough to influence global practices. Calls for the EU to adopt this approach in the biotechnology sphere highlight its potential,^68^ but widespread impact will depend on coordination among major economies.

On December 16, 2025, the European Commission proposed the EU Biotech Act, which establishes a comprehensive screening framework applicable to any person introducing SOCs or benchtop nucleic acid synthesis devices into the EU market.^69^ Any actor making listed sequences available in the EU must therefore verify the legitimacy of the customer and the stated end use. As a result, sellers—including those located outside the EU—would be required to function as security gatekeepers, responsible for assessing the buyer’s peaceful intent before a transaction may proceed. The proposal must still undergo the EU legislative process. If adopted, it would reflect the EU’s embrace of a market-access–based regulatory approach to biosecurity risks.

#### IV.B.4. Public Procurement Contracts (Hard Law)

The procurement model leverages government purchasing power to advance policy goals. By embedding conditions into funding contracts, governments can require recipients to comply with certain conditions.^70^ In the SNA context, this means publicly funded researchers may only procure nucleic acids from providers that conduct screening. The USA has already adopted this approach by restricting federally funded researchers to approved suppliers (see below, Section V).

The advantage of procurement rules is that they avoid imposing universal mandates upfront. Instead, they concentrate compliance within the large segment of providers serving publicly funded research. This levels the playing field among suppliers and creates powerful incentives for compliance when government demand is significant.

However, the reach of this model is inherently limited. In jurisdictions where public procurement represents only a small share of total demand, the impact will be marginal. Actors outside procurement chains remain unaffected, and private-sector markets continue operating without safeguards.

Thus, procurement or other funding contracts can be highly effective tool in jurisdictions with substantial public research funding, such as the USA, the European Union, or China. For providers, the commercial value of accessing these markets will outweigh any savings from bypassing screening.

#### IV.B.5. Liability (Hard Law)

Liability complements *ex ante* hard law by holding actors *ex post* legally responsible when they breach specific legal or contractual obligations or when their conduct falls below the standard of reasonable behavior (tort). In this way, liability—in theory—creates deterrence and incentivizes providers to comply with legal requirements as well as broader expectations of responsible practice. In the context of SNA, liability could arise under several branches of law:

- **Administrative law:** regulators may impose penalties for breaching statutory or regulatory biosecurity requirements.
- **Tort law**: injured parties may claim compensation for harm caused by negligent or reckless conduct. As Tucker notes, a gene-synthesis company could face liability under anti-terrorism or biosecurity laws if its DNA were used in a bioterrorist attack and the company failed to take precautions that a reasonable actor would have taken—eg rejecting an order with only a post office box as a contact.^71^
- **Contract law**: liability may arise if researchers breach procurement contracts.
- **Criminal law**: perpetrators may face prosecution when their conduct constitutes a violation of anti-terrorism or biosecurity laws.

In all of these cases, compliance with screening guidelines would arguably provide protection, as following them constitutes a proportional and reasonable safeguard.^72^

While liability can offer some deterrent effect, it has clear legal and policy limitations in this context.

To establish tort liability for negligence, claimants must satisfy core doctrinal requirements, including duty of care, breach, causation, and attribution. In the context of synthetic biology, these requirements are difficult to meet. Attribution may require tracing a specific nucleic acid sequence back to its source and establishing a clear chain of custody, which is often practically infeasible. Causation poses further challenges: courts are generally reluctant to impose liability where the immediate cause of harm is the intentional criminal act of a third party, which may be treated as an intervening or superseding cause.^73^ Even if a duty of care would be recognized, there remains substantial uncertainty as to the applicable standard of care that a court would expect a synthesis provider to meet in the absence of clear regulatory benchmarks.

Beyond these doctrinal constraints, liability also has inherent policy limitations. Tort law is fundamentally reactive, operating only after harm has occurred and therefore offering limited protection against low-probability but high-consequence events. In the case of a truly catastrophic outcome, potential damages would far exceed the assets of most actors in the SNA supply chain, rendering them effectively judgment-proof. As Shavell has shown, where actors cannot internalize the full social costs of harm, liability loses much of its deterrent force.^74^

For these reasons, liability should be understood as a supplementary safeguard, not as the primary mechanism for governing risks associated with SNAs. It underscores the need for *ex ante* regulatory obligations, rather than reliance on *ex post* litigation alone.

#### IV.B.6. Administrative Guidance (Soft Law)

Administrative guidance consists of documents issued by government ministries or agencies that reflect their interpretation of a law or regulation, or their current thinking on a topic. Although not legally binding, guidance clarifies statutory or regulatory requirements and outlines procedures. For example, agencies may issue guidance on the steps required to obtain market authorization or the format for submitting applications.^75^

The advantage of guidance is that it can be issued quickly and with fewer procedural hurdles than binding legislation. It is particularly well-suited for technical requirements, since it can be updated regularly and adapted to evolving risks. This flexibility makes it especially valuable for screening procedures, given that list-based approaches must be rapidly adjusted in response to the rapid advances in science, technology, and AI highlighted above. However, because guidance is not binding, compliance is uneven. Without monitoring or enforcement, firms may choose to disregard it, especially if compliance imposes costs.

Thus, administrative guidance is an effective complement to binding rules: it provides the flexibility and speed needed to keep pace with emerging challenges such as SOCs and AI. Its impact, however, depends on whether governments pair guidance with binding legislation and oversight and link it to liability protections.

#### IV.B.7. Ensuring compliance: Licensing and certification

Effective implementation of screening requirements requires mechanisms to ensure compliance. Two common approaches are licensing and certification.

***Licensing regimes*** require providers or users to obtain government authorization before entering the market. Licenses are typically conditional on meeting defined requirements and are monitored through audits or inspections.^76^ Applied to SNA, this could mean that sellers and users of synthetic DNA or benchtop devices are permitted to operate only with government approval. Scholars such as Leach have proposed licensing systems in the USA, including FBI background checks for buyers and sellers.^77^ Indeed, since high risk listed biological agents can only be transferred to government-approved users under existing biosecurity laws (see Section V below), the same requirement should apply, mutatis mutandis, to SNA.

***Certification schemes*** provide another means of ensuring compliance. Here, either governmental or non-governmental bodies accredit providers as meeting screening obligations,^78^ similar to certification systems for medical devices in the EU.^79^ Certification could also be developed internationally—drawing inspiration from models like the Forest Stewardship Council or PIC/S for pharmaceutical Good Manufacturing Practice (GMP) compliance.^80^ Reliance on foreign accreditation bodies is a further possibility. A user-based approach discussed above should be accompanied by a certification scheme; otherwise, compliance would be difficult to verify.

In sum, licensing and certification are not stand-alone solutions but complements to screening requirements.

#### IV.B.8. Conclusion: Promise and Limits of National Governance

In the absence of an international agreement, countries face a dilemma over whether to regulate SNAs. Many fear that doing so could hinder innovation or create a competitive disadvantage. In an ideal setting, international rules would be implemented through national law. Yet governments cannot remain idle in the meantime.

Countries with established or emerging bioeconomies pose the greatest risk—both domestically and through potential spillover effects across borders. They, therefore, carry responsibilities, to prevent transboundary harm. These states should therefore use their national governance tools to require screening, drawing on the range of policy instruments outlined above. Large market economies and governments that fund significant portions of research have particular leverage to create extraterritorial impact and should use it to promote screening compliance.

For countries with limited or no bioeconomies, screening will understandably be a lower priority. However, from a global biosecurity perspective, it remains essential to incentivize all states to adopt screening requirements in order to close the weakest-link problem.For an overview of national governance approaches, see Table 1.

The next section turns to private governance and its role alongside national and international governance.

### IV.C. Private Governance

Private governance refers to voluntary standards, codes of conduct, or best practices developed by companies or industry associations as a mode of self-regulation. These instruments are not legally binding but rely on reputational incentives and market pressures to encourage compliance. They have become increasingly common across globalized industries such as food or environmental products as a way to manage risks, align practices across jurisdictions, and signal accountability to customers.^81^ As will be shown below (Section V.D) international private standards developed by the IGSC have so far been the primary mechanism for regulating SNAs.

Private governance offers certain advantages: it can be developed quickly, adapt more easily to technological change, and draw directly on industry expertise. Importantly, it can also foster a culture of responsibility and serve as an interim measure while governmental frameworks remain underdeveloped.

However, the limits of self-regulation are clear. Voluntary standards cannot ensure universal compliance, since firms outside industry groups remain uncovered, and even members face no binding enforcement. Conflicts of interest also arise: profit-driven firms may prioritize commercial viability over safety. This article argues that for technologies with catastrophic potential—such as DNA synthesis—self-regulation can promote a culture of responsibility but cannot substitute for binding, enforceable regulation and legal oversight. For an overview of private governance approaches, see Table 3.

**Table 3:** Private governance approaches for regulating SNAs.

Type of governance	Main characteristics	Advantages for regulating SNA	Disadvantages/limitations
**1. Voluntary standards and codes of conduct (soft law/self-regulation)**	Industry-developed standards, guidelines, or best practices adopted voluntarily by companies or associations (eg, IGSC standards). Non-binding, reliant on peer or reputational pressure.	• Rapid to develop and update. • Draws directly on technical expertise of providers and scientific community. • Encourages global alignment of practices without waiting for formal regulation. • Builds a culture of responsibility and awareness within industry. • Can serve as an interim solution while legal frameworks evolve.	• Non-binding—no universal coverage or enforceability. • Firms outside industry associations remain unregulated. • Risk of uneven compliance and free-riding. • Vulnerable to conflicts of interest—profit motives may override safety concerns. • Lacks independent oversight and accountability mechanisms. .• Cannot address weakest-link problem globally
**2. Industry certification and auditing programs**	Voluntary accreditation schemes or third-party audits verifying compliance with industry standards.	• Provides some transparency and quality assurance. • Can complement national and international frameworks. • May improve customer and investor confidence.	• Participation voluntary and limited to motivated actors. • Enforcement and monitoring typically weak. • Can create a false sense of security if standards are superficial.

### IV.D. Conclusion: Governance Approaches

Taken together, international, national, and private governance each provide distinct tools for managing the global biosecurity risks of SNA.

- International agreement and harmonization remain the ideal, since only they can resolve the competitive disadvantage and weakest-link problem inherent in SNA regulation. Yet in practice, such consensus is difficult to achieve. Second-best approaches, such as international multi-stakeholder partnerships or the use of market leverage by major economies to create extraterritorial effects, are therefore important alternatives that could be pursued.
- Yet as long as there is no international action, national governments cannot stay idle. They have several regulatory tools they can use. Countries with developed or growing bioeconomies should act quickly, and developing countries should also take steps, as inaction could turn them into safe havens for misuse.
- While private governance is better than nothing, as it encourages some companies to screen, it is far from sufficient for managing risks as serious as those posed by SNAs.

The next section assesses how existing legal frameworks measure up against these governance approaches and findings.

## V. NUCLEIC ACID SYNTHESIS: ARE CURRENT LEGAL AND REGULATORY BIOSECURITY REGIMES FIT FOR PURPOSE?

This section turns from the normative to the descriptive: it surveys how current international, national, and private governance regulate SNA, and assesses whether they are fit for purpose in managing today’s biosecurity risks.

It is important to stress at the outset that biosecurity law and regulation are not new. Longstanding instruments—including the BWC, UN Security Council Resolution 1540, the IHR, and the pandemic agreement—as well as national measures such as select agent laws, export controls, and dual-use research regulations, were designed to prevent and mitigate biological risks. Collectively, they constitute the foundations of the current global biosecurity regime. However, these frameworks were adopted in a different technological era, one in which pathogen samples of *natural* origin were used and transferred. While a few jurisdictions have begun updating their regulatory frameworks to expressly include SNA within their scope, the vast majority have not.

**Diagram 4:**
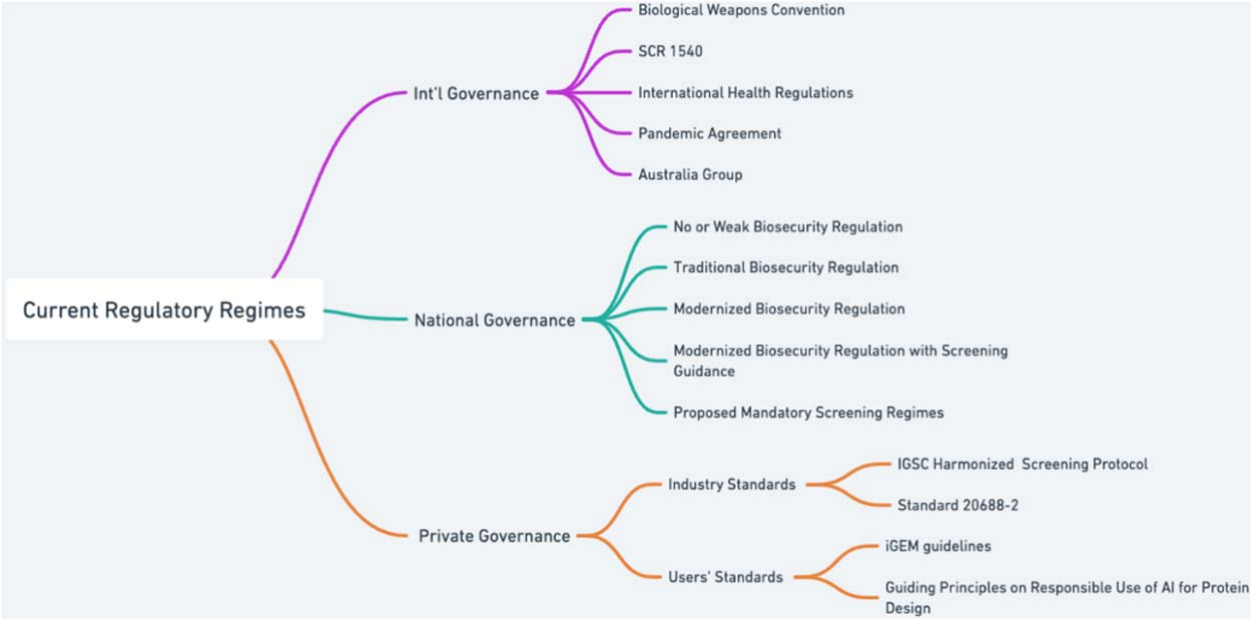
Current regulatory regimes.

Drawing on the governance framework, Diagram 4 below outlines the key legal and regulatory regimes. The analysis in this section follows this order.

Before proceeding with the analysis, it should be noted that this article does not address the regulation of AI models. As noted above, the AI–bio interface heightens biosecurity risks. This has prompted growing attention to potential regulatory approaches at the AI-model-development stage, including the use of technical guardrails, the assessment and moderation of model capabilities, and controls on access to high-risk models.^82^ While these issues are critically important, the regulation of AI models raises distinct legal and policy questions that fall outside the scope of this article.

### V.A. International Governance

As outlined above, international agreement represents the most effective approach for governing SNAs. The key question, then, is how existing international law compares against this ideal.

This section examines current international governance in two parts. The first consists of binding treaties. The second category comprises ‘club’ bodies.

#### V.A.1. International Treaties: Overview

Several international treaties lay out the foundations for biosecurity and pandemic prevention. These treaties require states to take measures to prevent international public health emergencies and biosecurity risks. The most important ones are the IHR, the BWC, the UNSCR 1540, and the recently negotiated pandemic agreement. Together, these instruments form the international legal regime for pandemic prevention and non-proliferation of biological weapons. Although none of these binding legal frameworks explicitly mention synthetic biology, international law is generally interpreted as technology-neutral, meaning its principles are applied irrespective of technological developments.^83^

Moreover, under the international rules on treaty interpretation (Articles 31–32 of the Vienna Convention of the Law of Treaties), treaties shall be interpreted ‘in good faith in accordance with the *ordinary meaning* to be given to the terms of the treaty in their context and in the light of its *object and purpose*’. As such, these international instruments, whose scope applies to diseases irrespective of origin or source and whose objective and purpose are for states to take measures to *prevent* the international spread of infectious diseases and the proliferation of biological weapons, should be interpreted, in an era of synthetic biology, to require states to take actions or adopt measures to prevent misuse of SNA.^84^

The following sections examine each of these international laws.

#### V.A.2. The International Health Regulations

The IHR is an international legal agreement with near-universal membership among states. The purpose of the IHR is to prevent the international spread of diseases,^85^ ‘irrespective of origin or source’.^86^ Thus, it applies to naturally occurring or man-made (accidental or deliberate) infectious diseases. As such, the IHR applies to SNA of pathogens that can cause infectious disease outbreaks. Further, the IHR requires all state parties to develop and maintain core public health capacities, including ‘the core capacities to prevent … events’ (Article 5).^87^ An ‘event’ is defined as ‘ a manifestation of disease or an occurrence that creates a potential for disease’. Thus, states should be understood as being under an obligation to establish core capacities to prevent outbreaks resulting from SNAs.

Further, as part of the IHR implementation process, the Joint External Evaluation (JEE)—a voluntary monitoring tool developed by the WHO—evaluates countries’ capacities to prevent, detect, and respond to public health threats.^88^ These threats may be natural, accidental, or deliberate in origin. Importantly, the JEE includes a dedicated focus on biosafety and biosecurity, with the stated goal of reducing dual-use risks, mitigating biological proliferation and deliberate use threats, and ensuring the safe transfer of biological agents.^89^ States are assessed based on whether they have enacted a comprehensive national biosafety and biosecurity regulatory framework that includes biosecurity monitoring activities.

Although the IHR or JEE do not explicitly mention synthetic biology or screening of synthesis orders, such activities, in line with the rules on treaty interpretation, should be understood as falling under the broader category of prevention of the international spread of disease, including mitigating deliberate use threats. To strengthen global preparedness, the JEE framework should be updated to include synthesis screening as a core component of biosecurity evaluation.

#### V.A.3. Pandemic Agreement

Although not yet in force, a new pandemic agreement has recently been adopted by WHO member states.^90^ This agreement establishes multilateral legal obligations related to the prevention, preparedness, and response to pandemics. The treaty deliberately does not distinguish between naturally emerging and man-made sources of pandemics, thereby effectively encompassing synthetic biology-related risks within its scope.

Article 4, which addresses the ‘prevention’ pillar, explicitly requires states to develop and strengthen their capacity to prevent the emergence or re-emergence of infectious diseases and manage biological risks. It also includes (Article 4(2) (i)) an obligation to develop or strengthen laboratory risk management, including *through biosafety and biosecurity training and practices*, and ensuring the safety and security of transportation.^91^

Given the potential for SNA to be misused in ways that could trigger pandemics, risks associated with SNA should be regarded as central to states’ prevention obligations under the agreement. In effect, this falls under the obligation to adopt biosecurity practices.

#### V.A.4. The Biological Weapons Convention and Security Council Resolution 1540

The BWC is a multilateral treaty prohibiting the development, production, and stockpiling of biological weapons. It is almost universal and has 188 state parties.^92^ The treaty defines biological weapons broadly as ‘microbial or other biological agents, or toxins, *whatever their origin or method of production*, of types … that have no justification for prophylactic, protective, or other peaceful purposes’ (Article 1). The text ‘whatever their origin or method of production’ clearly indicates that synthetically produced biological agents fall within the scope of the BWC. This has also been clarified by BWC Review Conferences.^93^

The BWC imposes a binding obligation of conduct on states: Article 3 prohibits the transfer of biological weapons to any recipient, and Article 4 says that they shall take any necessary measures to prohibit and prevent the development, production, stockpiling, acquisition, or retention of biological agents that might be used as weapons or for non-peaceful uses. These provisions are typically implemented at the national level through select biological agent laws and export controls. These laws (as explained in greater detail below) monitor the use, transfer, and export of selected, regulated biological agents. Since synthetic biological agents, as mentioned, fall within the scope of the BWC, such domestic measures should be updated to regulate the use, transfer, and export of SNAs.

General principles of international law establish that treaties are technology-neutral,^94^ and the BWC itself recognizes under Article 12 the need to account for scientific and technological developments. In today’s context, where SNA can be easily transferred, Article 4 should therefore be interpreted as requiring states to adopt ‘necessary measures’ to prevent their non-peaceful use.^95^ What was sufficient 50 years ago is no longer adequate; the content of ‘necessary and effective’ measures must evolve with emerging threats.^96^ In practice, this means adopting national rules that oblige providers to screen orders for both domestic and international transfers. Failure to do so exposes states to serious risks of misuse and may amount to non-compliance with their BWC obligations.^97^

In practice (as detailed below), most countries have not regulated the biosecurity risks associated with nucleic acid synthesis, and only a small number, most notably the USA and the UK, have adopted formal screening guidance. This widespread regulatory gap is, as argued, not consistent with the BWC, which is technology-neutral by design and is robust enough to cover synthetic biology. Member states of the BWC should make it clear, at meetings or periodic review conferences under Article 12, that complying with the Convention requires regulating nucleic acid synthesis.^98^ An interesting development in this context has been the ‘Draft specific and effective measures, including possible legally-binding measures, to strengthen and institutionalize the Biological Weapons Convention’ issued by the Working Group on the Strengthening of the BWC. It recommends ‘all States Parties to develop guidelines and regulations to manage the risks of possible intentional misuse of biology arising from emerging and converging technologies, such as artificial intelligence, synthetic biology, and quantum computing, filling a gap not currently covered by any other international processes’.^99^

Similarly, UNSCR 1540 imposes legally binding obligations on all UN member states to prevent the proliferation of nuclear, chemical, and biological weapons. Article 3 requires the adoption of national controls over the production, use, storage, and transfer of biological agents. Its terminology is aligned with the BWC, and as such, synthetic biological agents fall within its scope. It also mandates export control regimes and penalties for violations. Going forward, the resolution should be explicitly clarified as requiring SNA screening as part of effective domestic controls. Technical and capacity-building support provided under UNSCR 1540 should include assistance for states to establish and enforce such screening requirements.^100^

#### V.A.5. The Australia Group

Another relevant area of international governance is found in clubs, most notably, the Australia Group (AG). It is a group of 42 member states that was established to implement Article 4 of the BWC and UNSCR 1540. Its members include major economies such as India, Japan, and the Republic of Korea. Its purpose is to align national export controls to prevent biological and chemical weapons proliferation.^101^ Although the AG’s Guidelines and Commerce Control List (CCL), that is, the list of controlled goods, are non-binding, its influence is substantial, including beyond its membership. Many non-member countries, such as Singapore, have *de facto* aligned their national export regulations with AG guidelines and CCL.^102^

The AG CCL specifies the biological agents, toxins, and genetic elements that are subject to export restrictions.^103^ The *List of Human and Animal Pathogens and Toxins for Export Control* includes dangerous viruses, bacteria, toxins, and genetic material.^104^ Notably, the CCL covers not only natural pathogens and toxins but also their genetic elements, including synthetic elements.^105^In the case of bacteria and fungi, the controls extend to any genetic elements that ‘in themselves or through their transcribed or translated products represent a significant hazard to human, animal, or plant health, or could endow or enhance pathogenicity’.^106^

Thus, for SNA providers operating in AG member states, the CCL introduces legal obligations to apply for an export license when transferring regulated SNA internationally. While the AG does not explicitly mandate screening procedures, providers are effectively required to implement screening mechanisms to ensure compliance. In other words, to obtain the necessary licenses, providers must demonstrate that exported synthetic genetic elements do not violate applicable regulations. This results in a *de facto* requirement for sequence screening, making it an essential component of compliance for SNA providers within AG member states.

Despite the progress made by the AG in acknowledging the risks associated with synthetic biology, the CCL remains primarily focused on genetic elements of regulated pathogens. This approach, as mentioned above (Section III), is insufficient, as the reliance on predefined lists means that not all SOCs are covered.

Against this background, AG members should consider updating the export control list to encompass SOCs. To further strengthen compliance, AG members could collaborate on developing harmonized screening protocols to support providers in adhering to export controls. While such measures would be limited to export controls and cross-border transfers and would not cover domestic transfers, they would nonetheless provide an opportunity to introduce enhanced screening, and other non-member countries might follow them too.

#### V.A.6. Conclusion: Towards Modernizing International Governance

International law imposes clear obligations on states to adopt measures to prevent the outbreak of public health emergencies of international concern, pandemics, and the misuse of biological agents. While these instruments do not explicitly mention SNA, the general principle of technological neutrality applies. Moreover, under the rules on treaty interpretation, treaties need to be interpreted in line with their object and purpose. As such, in an age of synthetic biology, they should be understood as implying a legal duty to adopt effective measures that prevent the misuse of SNA.

To clarify states’ legal obligations in the context of synthetic biology, it is recommended that BWC member states, as well as other relevant international organizations such as the WHO—explicitly affirm the application of existing instruments to synthetic biology. In parallel, these international organizations should lead efforts to develop globally harmonized screening requirements that member states can adopt or draw upon in shaping their national regulations. Establishing clear, international harmonized standards would help close critical gaps in the current regulatory landscape and promote greater consistency worldwide.

Alongside treaties, informal ‘clubs’ such as the AG play an important role by harmonizing export controls. Although non-binding, the AG has significant influence, as many non-member states align their regulations with its guidelines. In practice, this creates a *de facto* requirement for sequence screening in international transfers. Yet the AG’s list-based approach remains outdated and fails to capture novel SOCs. Thus, members should expand export control regimes to cover SOC and make clear that this entails adopting screening requirements.

In sum, the international legal instruments needed to regulate SNA screening already exist—largely in line with what an ideal model laid out in Section IV would require. What remains is to clarify and operationalize these obligations so as to modernize them and make them fit for purpose.

### V.B. National Governance

This section examines whether, and in what ways, national governance has addressed the risks posed by SNA. It surveys national strategies, and the extent to which existing biosecurity laws have been modernized, highlighting both progress and gaps.

#### V.B.1 National Strategies for SNA Regulation

Most countries either lack biosecurity legal frameworks altogether or have only very limited ones, including concerning SNA. According to the 2021 Global Health Security Index (GHSI), which assesses 195 countries, over 100 countries have no biosecurity framework at all, and around 40 have extremely limited and meager ones.^107^ Among countries with biosecurity frameworks, national legal systems use a variety of approaches to regulate biosafety and biosecurity.^108^ Established legal systems include select biological agent laws, export controls, laboratory biosafety, regulations on responsible research (dual-use research of concern), and other laws. In practice, there are variations in the biosafety and biosecurity legal and regulatory frameworks among different countries.^109^

**Diagram 5:**
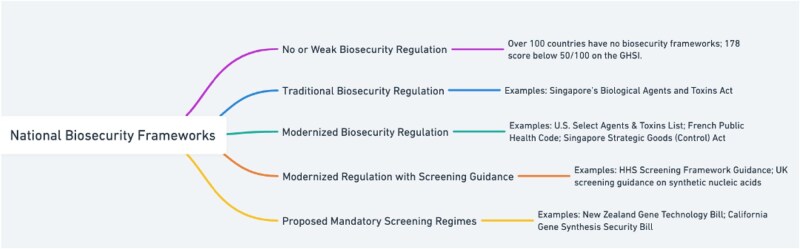
National biosecurity frameworks concerning SNAs.

Overall, five distinct regulatory approaches or strategies can be observed regarding the governance of the biosecurity aspects of SNA (see Diagram 5):

1. **No or Weak Biosecurity Regulation**: Captures the complete regulatory gap in many low and middle-income countries, such as Laos and Myanmar.^110^ The 2021 Global Health Security Index (GHSI) found that 178 countries score below 50 out of 100 on biosecurity^111^ and lack biosecurity laws.
2. **Traditional Biosecurity Regulation:** This category includes countries that have biosecurity-related laws and regulations, but these are traditional in scope, focusing on natural pathogens without addressing synthetic biology or synthetic genetic materials. Examples include Australia^112^ and the Republic of Korea.^113^
3. **Modernized Biosecurity Regulation:** Jurisdictions that have adapted legal frameworks to cover not only natural pathogens but also synthetic genes or pathogens. But have not, as of yet, adopted screening guidance. This includes Canada and France.^114^ It appears that China's 2020 Biosecurity Law also falls within this category.^115^
4. **Modernized Biosecurity Regulation with Screening Guidance:** In only two countries—the USA and the UK—governments have not only modernized their laws but have also adopted screening guidance to support compliance.^116^
5. **Proposed Mandatory Screening Regimes:** This is an emerging approach which pushes for mandatory screening. This approach is being considered in several bills, including in the European Union, New Zealand, and the USA Congress, but has not been adopted yet (see *Towards Binding Standards: Proposals for Mandatory Screening Laws* below).

Why do many countries lack regulatory biosecurity and screening frameworks? The reasons are varied and warrant further investigation beyond the scope of this article. However, based on current knowledge of national policymaking, contributing factors likely include a lack of political salience and awareness, insufficient advocacy, competing priorities, and concerns that regulation could hinder innovation and restrict the growth of the bioeconomy. Additionally, concerns about the financial burden of compliance, limited technical capacity, and a shortage of specialized expertise may also play a role.^117^ Also, research and development in synthetic biology is concentrated in a few countries; consequently, there is limited interest in regulating this field.^118^

The following sections examine two main regulatory approaches that have emerged for regulating SNAs: modernized regulation with screening guidance (category 4) and a mandatory screening approach (category 5).

#### V.B.2 Modernized Biosecurity Regulation with Screening Guidance

The GHSI highlights several countries with strong biosecurity legal and regulatory frameworks designed to prevent the accidental or deliberate misuse of biological agents. Notable examples include the USA, the United Kingdom, Australia, Canada, Germany, France, South Korea, Japan, and Singapore.^119^

However, of these, only a few countries have updated or modernized their frameworks—or at least parts of them—to explicitly cover SNA, not just natural pathogens. For example, France recently updated its regulation of hazardous microorganisms and toxins (MOT) under its Public Health Code; the regulated lists now include SNA alongside natural pathogens.^120^ However, France has not issued any guidance on how providers should screen orders, leaving a critical gap in practical implementation.

Further, in some countries, there is a gap between export control laws that incorporate the AG Common Control List—and therefore cover SNA—and their domestic laws. For example, in Singapore, the Strategic Goods (Control) Act follows the AG CCL and applies to SNAs, while the Biological Agents and Toxins Act, which governs domestic use and transfer, remains traditional and lists only natural pathogens.

The USA, ranked first of 195 countries by the GHSI index^121^, has the most comprehensive biosecurity legal framework, which also expressly regulates the risks associated with nucleic acid synthesis. Its legal system includes select agent laws and export controls, both of which apply not only to natural biological agents but also to the synthetic genetic parts of those agents. To further support compliance, the USA Health and Human Services (HHS) has issued screening guidelines targeted at the SNA industry and guidance targeted at users.

The UK has recently adopted a similar approach.^122^ It has a legal and regulatory framework for preventing the accidental or deliberate misuse of SNAs. This framework includes the Anti-terrorism, Crime and Security Act 2001, which (Schedule 5) notes that pathogens and toxins that could be used in an act of terrorism include SNAs.^123^ Further, following the adoption of the 2023 UK Biological Security Strategy, which outlines the UK's plan to build resilience against biological threats and position itself as a world leader in responsible innovation,^124^ in October 2024, it adopted the ‘UK screening guidance on SNA for users and providers.’^125^ As regards benchtop manufacturers, the UK guidance recommends that manufacturers build in screening functionalities, and that benchtop devices are only distributed to legitimate users. However, it is unclear how they plan to enforce this in practice.

Further, the EU is considering a new Biotech Act, and discussions within the EU have highlighted the need to manage responsible access to synthetic biology and regulate biosafety risks.^126^ The EU Commission also stressed that European standardization organizations must update outdated standards and develop new ones, especially regarding biomaterials and bio-based products.^127^

While awareness is growing in some countries and regions, regulation remains fragmented and outdated. This article examines the main features of the current US regulatory model, which, as the most advanced, can serve as a model or source of inspiration for countries looking to update their laws for the new era of synthetic biology. It highlights the model’s strengths and its shortcomings. The article next examines the US Select Agent Program, Export Controls, HHS Screening Guidance, and the Framework for Nucleic Acid Screening.

##### V.B.2.i. The US Federal Select Agent Program (FSAP)

The 2002 *Public Health Security and Bioterrorism Preparedness and Response Act*,^128^ requires the HHS to establish and regulate a list of biological agents and toxins that have the potential to pose a severe threat to public health and safety. The 2002 *Agricultural Bioterrorism Protection Act* requires the United States Department of Agriculture (USDA) to establish and regulate a list of biological agents that have the potential to pose a severe threat to animal health and safety, plant health and safety, or the safety of animal or plant products.^129^ These laws require the Centers for Disease Control and Prevention (CDC) and the USDA to review and republish the Select Agents and Toxins List (SATL).^130^

The SATL includes high-risk pathogens, including viruses such as smallpox.^131^ Importantly, the list applies to SNA of listed biological agents and toxins. Specifically, it applies to ‘nucleic acids that can produce infectious forms of any select agent virus’.^132^ And, ‘Synthetic nucleic acids that encode for the toxic form(s) of any toxins listed’.^133^ According to Centre for Disease Control (CDC) guidance, the regulation also includes virus sequences that are ‘inherently infectious and are immediate precursors to virus production’, even without additional exogenous factors.^134^ A key limitation of the FSAP is that its scope extends only to whole genomes, leaving SOCs outside its coverage.^135^

It is prohibited to possess, use, or transfer any select agent or toxin without a valid certificate of registration issued by the HHS Secretary.^136^ If a SNA provider receives an order for a sequence that falls under the SATL, the provider must be registered to possess and transfer it. The customer placing the order must be registered to possess the biological agent or toxin. Transfers are limited to licensed entities that are registered to possess, use, or transfer the specified agent or toxin.^137^

Given these legal requirements, SNA providers are effectively required to implement screening processes to identify regulated sequences. To support providers in fulfilling these obligations, the HHS has issued detailed screening guidance. This guidance will be discussed in further detail below.

##### V.B.2.ii. Export Controls: Export Administration Regulations and International Traffic in Arms Regulations

The Export Administration Regulations (EAR) are enforced by the US Department of Commerce and regulate the export of biological agents, including their genetic sequences. Oversight under the EAR is managed by the Bureau of Industry and Security (BIS), which classifies controlled items and includes them in the CCL.^138^

The CCL designates specific biological agents that are subject to export control due to their potential dual-use applications. Notably, the definition of controlled biological agents under the EAR is broad, covering not only natural pathogens but also synthetic genetic materials.^139^ The regulation explicitly applies to chromosomes, genomes, plasmids, transposons, and vectors (whether genetically modified or unmodified).^140^ A key limitation of the EAR is that its scope extends only to whole genomes, leaving SOCs outside its coverage.

Exporting these biological agents or their associated genetic sequences from the US requires obtaining an export license from the BIS. This licensing requirement supports the implementation of the BWC and AG mentioned above. The CCL also includes benchtop devices. As such, SNA providers and benchtop manufacturers must comply with the EAR by implementing screening mechanisms to identify regulated nucleic acids. The HHS Screening Guidance supports such compliance (see next). A further point to consider is whether SNA provided to foreign nationals within the US could be considered a ‘deemed export’ that requires licensing. If so, then this would suggest that customer due diligence is required also when providing DNA within the US.

However, several shortcomings must be highlighted: No license is required when shipping EAR-controlled biological agents (under ECCN 1C353) to AG members. Thus, not all exports are controlled equally. This is a biosecurity gap that would need to be addressed. A further shortcoming is that the oversight is list-based, meaning that other SOCs that are not listed can go undetected. Thus, in principle, users could circumvent the oversight by purchasing components and stitching them together or creating de novo SOCs.

Against this background, the International Traffic in Arms Regulations (ITAR) Category XIV (Toxins, Biological Agents, and Associated Equipment) could also play a role. Category XIV covers pathogens and biological agents that can be used in weapons, as well as their genetic elements.^141^ Its export controls are considerably stricter than those under the EAR, generally requiring a license for exports to any destination. Thus, ITAR can help close the gap left by the EAR. However, it too would have to be adapted to cover SOCs as well.

##### V.B.2.iii. The Department of HHS—‘Screening Framework Guidance for Providers and Users of Synthetic Nucleic Acids’

To help ensure compliance with the biosecurity legal framework addressed above, the US HHS has established a screening framework guidance.

In 2006, the US NSABB reviewed the adequacy of existing biosecurity laws and regulations in managing the risks of SNA. The findings, published in its report titled ‘*Addressing Biosecurity Concerns Related to the Synthesis of Select Agents*’ (2006 NSABB Report), recommended the development of specific screening guidance.^142^ Consequently, in 2010, HHS released the *Screening Framework Guidance for Providers of Synthetic Double-Stranded DNA* to address compliance with the SATL and the EAR. This was the first national governmental screening guidance to be published worldwide.^143^ This guidance recommended baseline measures: customer due diligence and sequence screening against the select agent lists (FSAP and CCL).

Due to rapid technological advancements highlighted above (Section III), the original guidance became outdated. The 2010 guidance had focused primarily on screening whole genomes of select agents, yet the ability to reconstruct such agents from multiple fragments, and the emergence of SOC underscored the need for more effective screening methods.

Consequently, HHS issued a revised version in 2023, titled ‘*Screening Framework Guidance for Providers and Users of Synthetic Nucleic Acids* (2023 HHS Guidance)**.** The updated guidance is broader in scope and makes several significant changes.^144^

(i) ***Regulated entities***: It is no longer addressed solely at providers, but also at manufacturers of benchtop nucleic acid synthesis devices, and users (eg, institutions, principal users, end users, and third-party vendors).^145^
(ii) ***From lists to SOC***: While previously it was recommended to screen strands using a ‘best match’ approach against regulated agents, providers are now encouraged to screen for SOCs, that pose a risk, even if no direct match is identified in existing databases, which are based on the regulated lists.^146^ Screening should include all ‘sequences that are recognized to contribute to pathogenicity or toxicity as information regarding these sequences and their verified functions and improved screening methods become available (or as feasible)’.^147^
(iii) ***Shorter sequences***: Under the previous 2010 guidance, 200 nucleotide sequences were compared against the SATL and CCL lists using a best-match algorithm. The 2023 HHS Guidance recommends that the length of screening be decreased to 50 nucleotides by 2026.
(iv) ***Benchtop devices:*** Manufacturers must screen customers and adopt mechanisms to monitor and verify the legitimate use of the devices.^148^
(v) ***End users***: The guidance requires end users to maintain comprehensive records of SNA orders and transfers. Users must also ‘develop and follow best practices in biosafety, biosecurity, and responsible conduct regarding the possession, use, and transfer of SOCs’.^149^

While the guidance is not legally binding, in practice, it carries weight as it reflects the expectations of the government agency concerning compliance with the SALT and EAR. As mentioned above, compliance with guidance could potentially protect from liability in case of harm.

Consequently, although the guidance is voluntary, most US-based nucleic acid synthesis providers have an incentive to comply. Whether they do in practice remains unclear. Some red teaming efforts suggest compliance is lacking.^150^ To improve this, an area for potential reform would be to introduce administrative enforcement mechanisms or strengthen liability. These are common in the pharmaceutical sector and could include inspections or audits to ensure compliance, license revocation or suspension for non-compliance, certification, fines and more.

##### V.B.2.iv. Framework for Nucleic Acid Synthesis Screening—White House Office of Science and Technology Policy (OSTP)

In April 2024 (revised in September 2024), following Executive Order 14110 on the Safe, Secure, and Trustworthy Development of AI,^151^ the White House Office of Science and Technology Policy (OSTP) released the Framework for Nucleic Acid Synthesis Screening.^152^ It incorporates and supplements portions of the 2023 HHS Guidance and its accompanying Companion Guide.

The framework applies to researchers who receive federal funding. It demands that, beginning on April 26, 2025**,** SNA and benchtop devices used for federally funded projects must be purchased exclusively from companies that comply with the Framework. In other words, federally funded researchers will be restricted to purchasing SNA from providers that conduct proper screening and from benchtop manufacturers that meet the required screening and oversight standards.^153^ This is significant because while no official figures on total federally funded research are publicly available, estimates suggest that roughly three-quarters of the US customer base for synthetic DNA consists of federally funded entities.^154^

Following the Presidential elections, Executive Order 14292 (May 5, 2025), ‘Improving the Safety and Security of Biological Research’,^155^ revoked EO 14110 and directed OSTP to revise the Framework by August 2025. Until then, the current Framework remains in force. At the time of writing, it has not been replaced yet.

In any event, the new Executive Order preserves the screening requirement and strengthens it. It states that the revised framework must ensure a commonsense approach while encouraging providers to adopt ‘comprehensive, scalable, and verifiable screening mechanisms’. All agencies funding life-science research are required to procure only from compliant providers, and non-compliance will be subject to stronger enforcement. The Order also extends beyond federally funded research by directing actions to promote screening in private and non-federal institutions as well.

In sum, as mentioned above, this public procurement approach creates a powerful market incentive: compliance with screening standards becomes a prerequisite for accessing federally funded research contracts and grants. Providers seeking to serve the US research community are therefore compelled by market forces to adopt screening practices.

#### V.B.3. Towards Binding Standards: Proposals for Mandatory Screening Laws

Seeking to strengthen comprehensive compliance, there have been some efforts to introduce legally binding screening mandates. To date, there are three notable examples of legislative proposals for mandatory screening: the California Gene Synthesis Security Bill (AB 70),^156^ the Securing Gene Synthesis Act introduced in the US Congress,^157^ the proposed EU Biotech Act,^158^ and the Gene Technology Bill.^159^ And just before going to print, a new Biosecurity Modernization and Innovation Act has been introduced in the US Congress. If enacted, these laws would represent the first legally binding screening requirements. However, given the concerns regarding innovation and competition, addressed above, no country has yet adopted mandatory screening.

##### V.B.3.i. California’s Gene Synthesis Security Bill (AB 70)

The California Gene Synthesis Security Bill (AB 70) is an example of state-level regulation seeking to influence global standards. Under this bill, companies that produce or sell SNA within California would be required to implement screening standards that are at least equivalent to those followed by members of the IGSC.

Additionally, the bill mandates that state universities and other institutions purchasing SNA must buy SNA only from providers that comply with screening standards. Given California’s prominent bioeconomy and its role as a major biotechnology hub, California has significant market leverage. For providers, losing access to the California market would be a commercial setback. As a result, even companies operating outside the US would face strong incentives to align their screening practices with California’s standards. Through this regulatory mechanism, California has the potential to influence global norms—even in the absence of comprehensive federal or international agreements.

Ultimately, however, the governor vetoed the bill, citing the costs of establishing a screening program and the need for oversight by the California Department of Public Health. He also pointed to fragmented global regulation as a disadvantage for California-based providers, questioning ‘whether a patchwork of state and federal regulations on biosecurity is the most effective way to approach an issue of international magnitude’.^160^

##### V.B.3.ii. The Securing Gene Synthesis Bill and the Biosecurity Modernization and Innovation Act

At the federal level, the proposed Securing Gene Synthesis Act (118th Congress, 2023–2024) conditions federal funding on compliance with screening standards. Under this bill, the HHS would be required to issue regulations requiring that federal agencies, as well as entities receiving federal funds, only purchase SNA from providers that adhere to approved screening protocols.^161^ Under the Biosecurity and Modernization Innovation Act, a bipartisan bill introduced in February 2026, mandatory SNA screening would be established.

##### V.B.3.iii. New Zealand Gene Technology Bill

The bill adopts a traditional ‘command and control’ regulatory approach towards screening. It gives power to issue a regulation on a screening mandate enforced by strict sanctions in case of noncompliance, including incorporating other national and/or international standards for screening. Screening obligations apply comprehensively to all SNA providers, benchtop device manufacturers, third-party vendors, and distributors.^162^ Non-compliance with these screening requirements is explicitly classified as a criminal offense under Clause 83, with offenders subject to prosecution under criminal law.

To ensure compliance, the regulator is granted significant enforcement powers, including the authority to obtain information and conduct inspections.^163^ This robust regulatory approach is likely to promote high levels of compliance with the screening requirements.

Additionally, Clause 149 authorizes the regulator to issue notices approving providers, manufacturers, third-party vendors, and distributors for the ‘purpose of the Bill’. This implies that only licensed entities would be permitted to operate within the sector. If this interpretation holds, it would give the regulator considerable control over who can offer SNA services. Such a system could enhance oversight, strengthen regulatory compliance, and improve biosecurity across the industry.

##### V.B.3.iv. The EU Biotech Act

In December 2025 the European Commission submitted its proposal for an EU Biotech Act.^164^ This Act would require providers of ‘biotechnology products of concern’, which include ‘sequences of concern’ and benchtop devices (to be listed in an Annex), to verify the ‘legitimate need’ and identity of prospective customers before making the good available. Sellers will be obliged to conduct a comprehensive assessment of the user's legitimacy by evaluating their professional credentials, as well as the existence of suitable facilities and biosecurity arrangements for the requested materials. Any transaction identified as suspicious must be refused, and the provider is legally obligated to report the attempt to a national contact point. To facilitate ongoing oversight, providers are required to maintain detailed records of these screenings and transactions.

#### V.B.4. Conclusion: Current National Governance Approaches

Currently, there are five distinct national regulatory approaches. Most countries do not have any biosecurity framework at all. Others have some biosecurity framework, but they are still traditional and do not address synthetic biology, let alone SOCs. Thus, the gap between the desired national approach outlined in Section IV and the current reality is stark and calls for reform.

In very few cases, we have identified three emerging approaches for regulating SNAs. The first is the USA/UK approach, which has expanded existing biosecurity laws to include synthetic biology and is supported by non-binding screening guidance. The second, still only proposed in several jurisdictions, involves mandatory screening laws. The third, adopted in the US, is to use procurement contracts to regulate users. Each of these approaches has its specific shortcomings, as outlined above.

### V.C. Private Governance

In the absence of national screening guidelines, several private initiatives have sought to fill the gap by adopting voluntary standards. The two most significant international efforts are the IGSC Harmonized Screening Protocol and ISO 20688-2. In addition, some researchers and users have adopted principles committing to procure genetic material only from companies that conduct screening, further reinforcing responsible practice.

#### V.C.1. IGSC Harmonized Screening Protocol

##### V.C.1.i. Establishment of the IGSC

In 2009, five leading gene synthesis companies established the IGSC to develop the first industry-led harmonized screening protocol,^165^ which has since been updated.^166^ As the SNA market grew, so did the IGSC. Today, it includes 36 companies that provide SNA or manufacture benchtop devices.^167^ Together, they make up about 80 per cent of the global market.^168^ IGSC accepts companies making over $5 million a year from gene synthesis as voting members. It also accepts small companies earning less than $5 million annually from gene synthesis and non-profit and academic institutions as non-voting members.^169^ While many providers aren't members of the IGSC, anyone can nonetheless follow the IGSC’s screening guidelines.

##### V.C.1.ii. The Screening Protocol

In September 2024, IGSC members adopted IGSC Harmonized Screening Protocol v3.0.^170^ It sets out best practices for sequence and customer screening for providers.^171^ The protocol is aligned with US regulations. It incorporates the 2023 HHS Guidance, the 2023 EO 14110, and the 2024 NSTC Framework. While the standard is a soft law instrument, in the absence of national guidance in most jurisdictions, it fills a regulatory void that other providers can rely on.

Gene length orders are screened against an IGSC ‘Restricted Pathogen Database’, which is an IGSC resource that aggregates global pathogen control lists (including data from US SATL and AG CCL, the EU list of dual-use items)^172^ and internationally coordinated reference databases (eg, NCBI/GenBank, EBI/EMBL, or DDBJ).^173^

The recent version seeks to address the recent technological challenges alluded to above. Thus, while the protocol requires screening of all sequences ≥200 base pairs, it is transitioning to screening ≥50 bp by 2026. Also, while no universal list of SOCs exists, the IGSC acknowledges this topic and will adapt its protocol as this field develops.

The screening process under the IGSC’s protocol is automated, is undertaken by the individual providers (or a party they contract with), but flagged orders undergo human review. If a sequence is deemed potentially hazardous, the provider must verify the legitimacy of the customer, ensuring they are affiliated with a recognized government laboratory, university, industrial laboratory, or other research institution.^174^ Customers are also screened against relevant lists of sanctions or denied party lists (eg, US OFAC’s SDN List, German HADDEX list).^175^

##### V.C.1.iii. Voluntary Nature and Impact

As a private standard, the IGSC screening protocol is not legally binding. It applies only to IGSC members. These include companies based in jurisdictions without screening requirements, yet they still conduct screening. For example, Genscript, headquartered in China, is an IGSC member and performs screening.^176^ However, IGSC members have incentives to comply, driven by peer accountability and concern for their reputations, especially since many are large companies for whom reputation is critical. That said, red-teaming tests have shown that not all IGSC members consistently screen orders.^177^ Without formal accountability mechanisms, there is always a risk of non-compliance. Another issue is that IGSC members only represent about 80 per cent of the global market, leaving a significant portion of providers, particularly those based in countries without national screening requirements, outside any oversight. The exact number of such providers is difficult to estimate. This gap underscores why international and national regulation remains essential.

#### V.C.2. ISO Standard 20688-2

ISO is an independent, non-governmental organization that brings global experts together as part of ISO Technical Committees to agree on private standards to encourage best practices, including biorisk management and biotechnology.^178^ In 2024, ISO issued a standard on screening. *ISO 20688-2: Biotechnology—Nucleic Acid Synthesis, Part 2: Requirements for the production and quality control of synthesized gene fragments, genes, and genome* sets standards for producing and controlling the quality of synthetic genes, gene fragments, and genomes.^179^ It is aimed at SNA producers, academic labs, and other users who need to evaluate the quality of SNAs.

While much of the standard focuses on production quality, like purity and yield, it also includes explicit biosafety and biosecurity requirements.^180^ Notably, providers must have ‘biorisk management’,^181^ which includes sequences and customer screening protocols. It expressly determines that to prevent the intentional or inadvertent misuse of DNA synthesis technologies and products, producers should make use of a DNA sequence screening mechanism.”^182^ This screening mechanism is set out in (see the box below with the text on Article 6.2. of the standard).

##### V.C.2.i. Screening Procedure

DNA sequences must be screened against known pathogens and toxin lists.^183^ To this end, the risk level of the synthetic genes needs to be assessed ‘according to the appropriate reference standard and documents of biosafety and biosecurity’. Annex G offers a sample risk ranking framework, referencing select agent lists like the AG CCL and part of the US SATL. Further, it determines that providers should screen for sequences longer than 50 bp and for SOC.^184^ SOC is defined as a sequence of 50 bp or greater that either encodes for biological functions or directly endows or enhances toxicity or pathogenicity.^185^ A shortcoming of this standard is that it applies only to DNA sequences and does not extend to RNA, even though many viruses of concern are RNA-based.

As regards customer screening, it determines that ‘the producer should have a procedure to ensure the legitimacy of customers, principal users, and end users of synthetic genes containing SOC’. Providers and third-party vendors of synthetic genes should know the customers’ end users and must notify them when their order contains SOCs.^186^

It should also be noted that an ISO standard specifically covering benchtop devices is under development.

**6.2 DNA Sequence Screening Mechanism**

All DNA producers should use a sequence screening mechanism to evaluate ordered sequences. This screening mechanism may be constructed in-house by producers or acquired from a third party. Screening systems can rely on an internationally recognized database of sequences of pathogen and toxin DNA and algorithms to screen ordered DNA sequences against that set of sequences. Screening should be conducted for sequences longer than 50 bp or in accordance with regional guidelines. If the screening system returns a hit for an ordered DNA sequence, the DNA producer shall choose whether to conduct follow-up screening or to reject the order. Where follow-up screening does not resolve concerns about an order, the producer may choose to refuse the order or to report the order to authorities, according to the particular case. For DNA producers that choose not to synthesize pathogen or toxin DNA, the synthesis should not proceed.

If a DNA producer chooses to synthesize pathogen or toxin DNA (ie, sequences that are hits according to their screening mechanism), the producer shall follow legitimate use guidelines. Evidence for legitimate use may include institutional affiliation, evidence of a legitimate research programme, customer publication history, or marketed products (eg, detection and test).

If a DNA producer chooses to synthesize pathogen or toxin DNA, the producer shall establish the corresponding capacity and facility for maintaining an appropriate control of biosafety and biosecurity.

When a customer orders DNA sequences from a regulated pathogen or toxin, the producer shall obtain a written description of the intended use for the synthetic DNA from the customer.

Whenever possible, the producer shall verify that the information obtained, including the intended use, is consistent with the customer’s activities. The result of the evaluation shall be documented.

It is recommended that producers document and retain for at least eight years the following information for orders about DNA sequences from a regulated pathogen or toxin:

(a) customer information (point-of-contact name, organization, address, email, and phone number);

(b) order sequence information (nucleotide sequences ordered, vector used); and

(c) order information (date placed and shipped, shipping address, receiver name).

(d) Intended use information (description from the customer, evaluation result).

***Box: ISO Sequence Screening Mechanism.***

##### V.C.2.ii. Voluntary nature and impact

ISO standards are private and voluntary. However, because ISO typically develops standards for highly technical areas, governments often adopt them by reference in their laws and regulations. Lacking the expertise, time, or capacity to develop their standards, many governments rely on ISO. An ISO standard on screening could support countries that lack the resources to develop their frameworks but urgently need them. It would be particularly valuable for countries aiming to grow their bioeconomies.

#### V.C.3. Self-Regulation by Users

There have also been initiatives by users to self-impose restrictions, committing to purchase SNA only from companies that conduct screening*.* The *Guiding Principles on Responsible Use of AI for Protein Design* determine that researchers will obtain DNA synthesis services from providers that demonstrate adherence to industry-standard biosecurity screening practices.^187^ Similarly, iGEM guidelines on responsible synthetic biology require all AI-generated DNA sequences ordered by iGEM teams to be screened and documented.^188^  *The WHO Global Guidance Framework for the Responsible Use of the Life Sciences: Mitigating Biorisks and Governing Dual-Use Research* also reaffirms that the responsibility of all users, along with their affiliated institutions, is to contribute to the safe and responsible conduct of research involving potentially dangerous materials.^189^

#### V.C.4. Conclusion

International private standards have tried to fill the regulatory screening gap. Companies, unsure how to comply with select agent laws and export control requirements, developed the IGSC screening framework to harmonize their practices. Similarly, the ISO standard is another private sector attempt to establish clear standards. Companies that follow these standards are seen as adhering to quality biotechnology or biosecurity practices, which are critical for businesses marketing to buyers concerned with biosecurity. Researchers are also adopting non-binding principles regarding the ordering of SNA.

But these standards are voluntary, and it remains unclear how many providers and users around the world comply with them. That’s the core issue: private standard setting helps, but it isn’t enough. Without government-backed rules and enforcement, widespread compliance and consistent biosecurity practices remain out of reach.

## VI. CONCLUSION AND GENERAL RECOMMENDATIONS

SNA create significant biosecurity risks that demand effective legal and regulatory oversight. This article has examined how such regulation should be designed in light of the unique characteristics of SNA: it is transnational in nature, subject to weakest-link vulnerabilities, potentially catastrophic in impact, and technically complex—especially amid rapid advances in AI.

Ideally, to enhance global biosecurity, SNA should be governed through a binding international agreement supported by harmonized international standards developed with the participation of relevant stakeholders. Without such an agreement, most states will remain reluctant to adopt mandatory screening requirements. The gap between this ideal and the current reality—still dominated by voluntary private standards and a few national guidance documents—is stark and underscores the urgency of multi-level legal reform.

### VI.A. International Law

The international legal instruments necessary to regulate SNA screening already exist. Instruments such as the BWC, UN Security Council Resolution 1540, the IHR and pandemic agreement already impose obligations on states to prevent the spread of infectious diseases and the misuse of biological agents. To clarify and modernize these obligations:

- BWC member states should explicitly clarify this issue.
- The BWC and WHO could serve as platforms for developing internationally harmonized screening standards to guide national implementation.

### VI.B. Australia Group

Members of the AG should expand their export control lists to include SOCs and collaborate on developing harmonized screening procedures. Given the AG’s global influence, even non-member states often align their laws with its guidelines, making it a powerful vehicle for spreading best practices.

### VI.C. National Law

Most countries lack comprehensive biosecurity legislation. Where frameworks exist, they typically regulate natural pathogens rather than SNAs. To modernize national governance:

- Update laws to explicitly cover SNAs, including SOCs, and clarify screening obligations for providers.
- Select regulatory tools suited to each national context—such as mandatory screening, procurement, or guidance.
- Close domestic gaps: many countries strictly control exports of pathogen sequences but not domestic transfers.
- Issue technical guidance—either nationally or by reference to international models such as US, UK, or ISO standards—to operationalize screening obligations.

For countries with established or emerging bioeconomies, these reforms are urgent regardless of international progress. Others should adopt baseline screening rules to avoid becoming regulatory safe havens. Clarifying BWC obligations will further provide the framework for coordinated national implementation.

### VI.D. Market Leverage and Funding Incentives

Large market economies and major research funders have the capacity to drive global practice. By conditioning market access, procurement eligibility, and funding on adherence to screening requirements, they can create powerful incentives that set *de facto* global norms and accelerate adoption beyond their borders.

### VI.E. Coalitions and Clubs

In the absence of universal agreement, coalitions of willing states and multi-stakeholder partnerships can make meaningful progress. These coalitions could build upon existing ‘club’ models such as the AG, or others like North Atlantic Treaty Organization (NATO), to align screening practices.

### VI.F. Technical and Stakeholder Engagement

The technical challenges of screening—such as identifying SOCs, managing short-fragment synthesis, and integrating AI tools—underscore the need for continuous engagement with the scientific community and industry. Stakeholder participation is essential to ensure that regulatory measures remain scientifically relevant.

In conclusion, SNA present a global catastrophic risk that demands global regulation. Current regulation is far from ideal. The good news is that achieving better oversight does not require inventing new international systems from scratch; instead, it calls for building on and modernizing the frameworks we already have. Biosecurity as a principle and in concrete requirements is already embedded in international law and in many national laws and regulations. But most regulatory frameworks haven’t kept pace with advances in biotechnology, especially nucleic acid synthesis. The task is clear: bring current biosecurity laws and regulations—both international and national—into alignment with today’s biotechnologies so innovation can proceed securely.

## Footnotes

1 Jacob Beal & Tessa Alexanian, *Creating Enforceable Biosecurity Standards for Nucleic Acid Providers*, 9 Engineering Biology 1 (2025); Forrest W. Crawford et al., *Securing Commercial Nucleic Acid Synthesis*, 12 Rand. Health Q. 4 (2024), https://pmc.ncbi.nlm.nih.gov/articles/PMC11630105/; T. Trond Arne Undheim, *The Whack-a-Mole Governance Challenge for AI-Enabled Synthetic Biology: Literature Review and Emerging Frameworks*, 12 Front. Bioeng. Biotechnol. 1359768 (2024), https://www.frontiersin.org/articles/10.3389/fbioe.2024.1359768/full

2 Worldwide Strategies, https://biooekonomie.de/en/topics/in-depth-reports-worldwide [accessed on 2 March 2026].

3 Crawford et al., *supra* note 1.

4 Jee Loon Foo et al., *Establishing Chromosomal Design-Build-Test-Learn Through a Synthetic Chromosome and Its Combinatorial Reconfiguration*, 3 Cell Genom. 100435 (2023), https://doi.org/10.1016/j.xgen.2023.100435 Ana Sofia Oliveira et al., *Spent Yeast Valorization for Food Applications: Effect of Different Extraction Methodologies*, 11 Foods 4002 (2022), https://doi.org/10.3390/foods11244002.

5 Shuai Xue et al., *A Synthetic-Biology-Inspired Therapeutic Strategy for Targeting and Treating Hepatogenous Diabetes*, 25 Mol. Ther. 443 (2017), https://doi.org/10.1016/j.ymthe.2016.11.008.

6 Michael R. Connor & Shota Atsumi, *Synthetic Biology Guides Biofuel Production*, 2010 J. Biomed. Biotechnol. 541698 (2010), https://doi.org/10.1155/2010/541698.

7 Worldwide Strategies, *supra* note 2; For example, U.K. National Vision for Engineering Biology (2023), https://www.gov.uk/government/publications/national-vision-for-engineering-biology/national-vision-for-engineering-biology [accessed on 2 March 2026]. Thailand is emerging as Southeast Asia’s prime destination for biotechnology development, https://www.boi.go.th/upload/content/Thailand%20as%20prime%20destination%20for%20biotech%20business%20Final_36306.pdf [accessed on 2 March 2026]. The Second Semiconductor Myth with Biotechnology ‘... Government Announces’ Advanced Bio Initiative (South Korea), https://www.news1.kr/it-science/general-science/5396941 [accessed on 2 March 2026]. South Korea to Launch National Bio Committee in December to Boost Bio Industry, https://www.businesskorea.co.kr/news/articleView.html?idxno=228474#google_vignette [accessed on 2 March 2026].

8 Braden R. Leach, *The Code of Life and Death*, 4 Notre Dam J. Emerging Tech*.* 44 (2023).

9 Transparency Market Research, *Gene Synthesis Market: Global Market by Method, Service Type, Application, End-User & Region, Forecast to 2033* (2023), https://www.transparencymarketresearch.com/gene-synthesis-market.html. [accessed on 2 March 2026].

10 National Academies Sciences Engineering Medicine, *Biodefense in the Age of Synthetic Biology* (2018), https://nap.nationalacademies.org/catalog/24890/biodefense-in-the-age-of-synthetic-biology [accessed on 2 March 2026]; Biological Weapons Convention Recent Advances in Gene Editing and Synthesis Technologies and their Implications, Submitted by the United States of America; Meeting of the States Parties to the Convention on the Prohibition of the Development, Production and Stockpiling of Bacteriological (Biological) and Toxin Weapons and on Their Destruction (2018), BWC/MSP/2018/MX.2/WP.5; James Revill & Catherine Jefferson ‘*Tacit Knowledge and the Biological Weapons Regime*’ 41 Sci. Publ. Policy 597 (2014).

11 Biosafety concerns the containment principles, technologies, measures, and practices that are implemented to prevent *unintentional* exposure to biological agents or their *inadvertent* or *accidental* release. Examples of biosafety risks include dual-use research on pathogens with pandemic potential, such as vaccine of gain of function studies, which could result in accidental and unintentional release of dangerous pathogens or toxins. Biosecurity concerns the principles, technologies, measures, and practices that are implemented to prevent unauthorized access, loss, theft, misuse, diversion, or release of biological agents WHO, *Global Guidance Framework for the Responsible Use of the Life Sciences: Mitigating Biorisks and Governing Dual-Use Research* (2022).

12 Volker Thiel, *Synthetic Viruses—Anything New?*, 14 PLoS Pathog. e1007019 (2018), https://doi.org/10.1371/journal.ppat.1007019.

13 WHO, *supra* note 11; U.S. Department of Health & Human Services, *Screening Framework Guidance for Providers and Users of Synthetic Nucleic Acids* (2023), https://aspr.hhs.gov/S3/Pages/Synthetic-Nucleic-Acids.aspx.; J. Craig Venter et al., *Synthetic Chromosomes, Genomes, Viruses, and Cells*, 185 Cell 1 (2022), https://doi.org/10.1016/j.cell.2022.06.046.

14 B. D. Trump, *Synthetic Biology Regulation and Governance: Lessons from TAPIC for the United States, European Union, and Singapore*, 121 Health Policy 1139–1146 (2017).

15 Roberta Kwok, *Five Hard Truths for Synthetic Biology*, 463 Nature 288 (2010), https://pubmed.ncbi.nlm.nih.gov/20090726/.

16 Crawford et al., *supra* note 1.

17 Cabinet Office, *UK Biological Security Strategy, CP 858* (June 12, 2023), https://www.gov.uk/government/publications/uk-biological-security-strategy; [accessed on 2 March 2026]. National Biodefense Strategy and Implementation Plan for Countering Biological Threats, Enhancing Pandemic Preparedness, and Achieving Global Health Security, *The White House* (Oct. 2022), https://bidenwhitehouse.archives.gov/wp-content/uploads/2022/10/National-Biodefense-Strategy-and-Implementation-Plan-Final.pdf [accessed on 2 March 2026]. Gemma Bowsher, A Multifaceted Threat, in Essentials of Biological Security: A Global Perspective (Ryan N. J. Calder ed., 2023), https://doi.org/10.1002/9781394189045.ch3; Sarah R. Carter et al., *The Convergence of Artificial Intelligence and the Life Sciences: Safeguarding Technology, Rethinking Governance, and Preventing Catastrophe*, Nucl. Threat Initiative (2023), https://www.nti.org/wp-content/uploads/2023/10/NTIBIO_AI_FINAL.pdf [accessed on 2 March 2026]. Nicole E. Wheeler et al., *Progress and Prospects for a Nucleic Acid Screening Test Set*, 29 Appl. Biosaf*.* 133 (2024), https://doi.org/10.1089/apb.2023.0033.

18 J. Dauparas et al., *Robust Deep Learning–Based Protein Sequence Design Using ProteinMPNN*, 378 Science, Sept. 15, 2022, at 49–56, https://doi.org/10.1126/science.add2187.

19 Nuclear Threat Initiative, Statement on Biosecurity Risks at the Convergence of AI and the Life Sciences, *NTI* (July 17, 2025), https://www.nti.org/analysis/articles/statement-on-biosecurity-risks-at-the-convergence-of-ai-and-the-life-sciences/; [accessed on 2 March 2026]. Josie Barton & Stewart Patrick, *Mitigating Risks from Gene Editing and Synthetic Biology: Global Governance Priorities, Carnegie Endowment for International Peace* (Oct. 2024), https://carnegieendowment.org/research/2024/10/mitigating-risks-from-gene-editing-and-synthetic-biology-global-governance-priorities?lang=en [accessed on 2 March 2026]; Carter et al., *supra* note 17; and Stephanie Batalis et al., *Safeguarding Mail-Order DNA Synthesis in the Age of Artificial Intelligence*, 29 Appl. Biosaf. 79–84 (June 20, 2024), https://doi.org/10.1089/apb.2023.0020. The National Academies of Sciences, Engineering, and Medicine, *The Age of AI in the Life Sciences: Benefits and Biosecurity Considerations* (Nat’l Acad. Press 2025) (review), https://doi.org/10.17226/28868 [accessed on 2 March 2026].

20 Carter et al., *supra* note 17. Douglas Robinson & Daniel Nadal, *Synthetic Biology in Focus: Policy Issues and Opportunities in Engineering Life*, OECD Sci. Tech. & Indus. Working Paper No. 2025/03 (Feb. 24, 2025), OECD, https://www.oecd.org/content/dam/oecd/en/publications/reports/2025/02/synthetic-biology-in-focus_42893a6a/3e6510cf-en.pdf, 19 [accessed on 2 March 2026].

21 Smriti Mallapaty, *New Virus-Genome Website Seeks to Make Sharing Sequences Easy and Fair*, Nature (Sept. 9, 2024), https://www.nature.com/articles/d41586-024-02864-x.

22 Global Innovation Index 2023, https://www.wipo.int/global_innovation_index/en/2023/; Nicole E. Wheeler et al., *Developing a Common Global Baseline for Nucleic Acid Synthesis Screening*, 29 Appl. Biosaf. 71 (2024), https://doi.org/10.1089/apb.2023.0034.

23 Sarah R. Carter et al., *Benchtop DNA Synthesis Devices: Capabilities, Biosecurity Implications, and Governance, Nuclear Threat Initiative* (May 2023), https://www.nti.org/wp-content/uploads/2023/05/NTIBIO_Benchtop-DNA-Report_FINAL.pdf [accessed on 2 March 2026].

24 James Randerson, *Did Anyone Order Smallpox?*, The Guardian (June 23, 2006), https://www.theguardian.com/science/2006/jun/23/weaponstechnology.guardianweekly. [accessed on 2 March 2026].

25 Kevin M. Esvelt, *It Shouldn't Be Easy to Buy Synthetic DNA Fragments to Recreate the 1918 Flu Virus*, MIT Media Lab (May 8, 2024), https://www.media.mit.edu/articles/it-shouldn-t-be-easy-to-buy-synthetic-dna-fragments-to-recreate-the-1918-flu-virus/. [accessed on 2 March 2026].

26 World Economic Forum & Nuclear Threat Initiative, *Biosecurity Innovation and Risk Reduction: A Global Framework for Accessible, Safe and Secure DNA Synthesis* (2020), https://www.nti.org/wp-content/uploads/2020/01/Biosecurity_Innovation_and_Risk_Reduction.pdf; [accessed on 2 March 2026]. Max Langenkamp, *Securing Benchtop DNA Synthesizers: As the field of Benchtop DNA synthesis evolves, it will require ongoing monitoring*, Biotechnology (Institute for Progress, 10 Dec. 2024), https://ifp.org/securing-benchtop-dna-synthesizers/. [accessed on 2 March 2026].

27 Piers Millett et al., *Beyond Biosecurity by Taxonomic Lists: Lessons, Challenges, and Opportunities*, Health Secur., (2023), at 521, https://doi.org/10.1089/hs.2022.0109; ​G. D. Godbold et al., *Categorizing Sequences of Concern by Function To Better Assess Mechanisms of Microbial Pathogenesis* 90 Infect. Immun. e00334-21 (2022), https://doi.org/10.1128/IAI.00334-21.

28 U.S. Department of Health & Human Services, *supra* note 13; Wheeler et al., *supra* note 22.

29 World Economic Forum & Nuclear Threat Initiative, *supra* note 26; Stefan A. Hoffmann et al., *Safety by Design: Biosafety and Biosecurity in the Age of Synthetic Genomics*, 26 iScience 107064 (2023), https://doi.org/10.1016/j.isci.2023.107064.

30 Engineering Biology Research Consortium, *Strengthening a Safe and Secure Nucleic Acid Synthesis Ecosystem: Outcomes of EBRC Stakeholder Engagement* (Jan. 2025), https://ebrc.org/wp-content/uploads/2025/02/EBRC-2025-Strengthening-a-Safe-and-Secure-Nucleic-Acid-Synthesis-Screening-Ecosystem.pdf; [accessed on 2 March 2026]. International Biosecurity and Biosafety Initiative for Science (IBBIS), *Common Mechanism* (updated Jul. 2025), https://ibbis.bio/our-work/common-mechanism/. [accessed on 2 March 2026].

31 Matthew Hutson, *Biothreat Hunters Catch Dangerous DNA before It Gets Made*, Nature (Oct. 2, 2025), https://doi.org/10.1038/d41586-025-03230-1, [accessed on 2 March 2026]. Bruce J. Wittmann, et al., *Strengthening Nucleic Acid Biosecurity Screening against Generative Protein Design Tools*, 390 Science, Oct. 2, 2025, at 82–87, http://dx.doi.org/10.1126/science.adu8578.

32 Jacob Beal & Tessa Alexanian, *Creating Enforceable Biosecurity Standards for Nucleic Acid Providers*, Engineering Biology 9(1) (2025).

33 Engineering Biology Research Consortium, *supra* note 30, https://ebrc.org/wp-content/uploads/2025/02/EBRC-2025-Strengthening-a-Safe-and-Secure-Nucleic-Acid-Synthesis-Screening-Ecosystem.pdf; [accessed 2 March 2026]. Stephanie Batalis et al., *Safeguarding Mail-Order DNA Synthesis in the Age of Artificial Intelligence*, 29 Appl. Biosaf. 79–84 (June 20, 2024), https://doi.org/10.1089/apb.2023.0020; Curtis Matthew Sharkey et al., *Enhancing Gene Synthesis Security: An Updated Framework for SNAScreening and the Responsible Use of Synthetic Biological Materials*, 29 Appl. Biosaf. 63 (2024), https://doi.org/10.1089/apb.2023.0036.

34 David Baker & George Church, *Protein Design Meets Biosecurity*, 383 Science 349 (25 Jan. 2024).

35 Curtis Matthew Sharkey et al., *supra* note 33, https://doi.org/10.1089/apb.2023.0036.

36 Wheeler et al., *supra* note 22, 71–78.

37 Sarah R. Carter et al., *supra* note 23.

38 Sarah R. Carter et al., *supra* note 23, at 27–29.

39 Moody’s, *Four Requirements for Customer Due Diligence for Banks (online article)*, Moody’s (6 May 2025), https://www.moodys.com/web/en/us/kyc/resources/insights/four-requirements-cdd-for-banks.html. [accessed 2 March 2026].

40 Exec. Order No. 14110, 88 Fed. Reg. 75,191 (Oct. 30, 2023)

41 WHO, *supra* note 11.

42 Responsible AI x Biodesign, https://responsiblebiodesign.ai/ [accessed 2 March 2026]; ​Ewen Callaway, ‘*Could AI-Designed Proteins Be Weaponized? Scientists Lay Out Safety Guidelines*’, Nature, (Mar. 8, 2024), at 478, https://www.nature.com/articles/d41586-024-00699-0.

43 iGEM, *Responsibility Conference 2.0 Reports* (Nov. 2023), https://static.igem.org/websites/jamboree/2023/downloads/responsibility-conference-2-0-reports.pdf. [accessed 2 March 2026].

44 Steph Batalis & Vikram Venkatram, *Breaking Down the Biden AI EO: Screening DNA Synthesis and Biorisk*, Center for Security and Emerging Technology (Nov. 16, 2023), https://cset.georgetown.edu/article/breaking-down-the-biden-ai-eo-screening-dna-synthesis-and-biorisk/; Leach, *supra* note 8.

45
Joost Pauwelyn, Ramses Wessel & Jan Wouters eds., Informal International Lawmaking (Oxford Univ. Press 2012). Ayelet Berman et al., eds., Informal International Lawmaking: Case Studies (Oxford Univ. Press 2012). Sabino Cassese et al., eds., Global Administrative Law: The Casebook (3^rd^ ed. RPA/IILJ 2012)

46
Lester M. Salamon, The Tools of Government: A Guide to New Governance 117–45 (Oxford Univ. Press 2002).

47
Ian Ayres & John Braithwaite, Responsive Regulation: Transcending the Deregulation Debate (Oxford Univ. Press 1992).

48 Kenneth W. Abbott & Duncan Snidal, as in Kenneth W. Abbott & Duncan Snidal, ‘*Strengthening International Regulation Through Transnational New Governance: Overcoming the Orchestration Deficit*’, 42 Vand. J. Transnat’l Law 501–578 (2009); Anne-Marie Slaughter, A New World Order (Princeton Univ. Press 2004); Kenneth W. Abbott et al., eds., International Organizations as Orchestrators (Cambridge Univ. Press 2015).

49 Ayelet Berman, *Is There a Stagnation in International Law?*, 109 Proc. Am. Soc’y Intl. L. 71 (2015).

50
Joost Pauwelyn et al., Rethinking Participation in Global Governance: Voice and Influence After Stakeholder Reforms in Global Finance and Health (Oxford Univ. Press 2022); Jonas Tallberg et al., The Opening Up of International Organizations: Transnational Access in Global Governance (Cambridge Univ. Press 2013).

51 Ayelet Berman, *supra* note 49.

52 Pauwelyn et al., *supra* note 50.

53 Slaughter, *supra* note 48.

54 Ayelet Berman, *Between Participation and Capture in International Rule-Making: The WHO Framework of Engagement with Non-State Actors*, 32 Eur. J. Int’l L. 227 (2021), https://doi.org/10.1093/ejil/chab014.

55 Ayelet Berman, *Industry, Regulatory Capture and Transnational Standard Setting*, 111 AJIL Unbound 226 (2017).

56 Ayelet Berman, *The Rise of Multistakeholder Partnerships*, 111 Proc. Ann. Meeting Am. Soc’y Int’l L. 205 (2017), https://www.jstor.org/stable/26628034. [accessed 2 March 2026].

57 National Science Advisory Board for Biosecurity, *Addressing Biosecurity Concerns Related to the Synthesis of Select Agents* (Dec. 2006), archived by the Federation of American Scientists, biosecurity.fas.org/resource/documents/NSABB%20guidelines%20synthetic%20bio.pdf. [accessed 2 March 2026]. Steph Batalis & Vikram Venkatram, *Breaking Down the Biden AI EO: Screening DNA Synthesis and Biorisk, Center for Security and Emerging Technology* (Nov. 16, 2023), https://cset.georgetown.edu/article/breaking-down-the-biden-ai-eo-screening-dna-synthesis-and-biorisk/. [accessed 2 March 2026].

58 World Economic Forum & Nuclear Threat Initiative, *supra* note 26; International Gene Synthesis Consortium, *Harmonized Screening Protocol v3.0* (2024), https://genesynthesisconsortium.org/wp-content/uploads/IGSC-Harmonized-Screening-Protocol-v3.0-1.pdf. [accessed 2 March 2026].

59 Centre for Long-Term Resilience, *SNAScreening: Overcoming Challenges with Implementation* (2023), https://www.longtermresilience.org/wp-content/uploads/2024/09/Synthetic-Nucleic-Acid-Screening-Overcoming-challenges-with-implementation-Website-copy-1.pdf. [accessed 2 March 2026].

60
Organisation for Economic Co-operation and Development, Risk and Regulatory Policy: Improving the Governance of Risk, OECD Reviews of Regulatory Reform (Paris: OECD Publishing 2010), https://doi.org/10.1787/9789264082939-en.

61 Centre for Long-Term Resilience, *supra* note 59.

62 Salamon, *supra* note 46, chapter 5. Daniel H. Cole & Peter Z. Grossman, *When Is Command-and-Control Efficient? Institutions, Technology, and the Comparative Efficiency of Alternative Regulatory Regimes for Environmental Protection*, 1999 Wis. L. Rev. 887 (1999).

63 Sarah R. Carter & Robert M. Friedman, *DNA Synthesis and Biosecurity: Lessons Learned and Options for the Future*, J. Craig Venter Institute (2015), https://www.jcvi.org/research/dna-synthesis-and-biosecurity-lessons-learned-and-options-for-the-future. [accessed on 2 March 2026].

64 Stefan A. Hoffmann et al., *Safety by Design: Biosafety and Biosecurity in the Age of Synthetic Genomics*, 26 iScience art. 106165 (Mar. 17, 2023), https://doi.org/10.1016/j.isci.2023.106165; [accessed on 2 March 2026]. Bridget Williams & Rowan Kane, *Preventing the Misuse of DNA Synthesis*, Institute for Progress (Feb. 15, 2023), https://ifp.org/preventing-the-misuse-of-dna-synthesis/. [accessed on 2 March 2026].

65 Int’l Law Comm’n, Draft Articles on Prevention of Transboundary Harm from Hazardous Activities, with Commentaries, in *Report of the International Law Commission on the Work of Its Fifty-Third Session*, U.N. GAOR, 56th Sess., Supp. No. 10, at 148, U.N. Doc. A/56/10 (2001), reprinted in [2001] 2 Y.B. Int’l L. Comm’n 148, U.N. Doc. A/CN.4/SER.A/2001/Add.1 (Part 2).

66
Uzair H. Ghori, Export Restrictions and Export Controls: From WTO to the Realm of Global Security (Edward Elgar Publ’g Ltd 2023).

67
Anu Bradford, The Brussels Effect: How the European Union Rules the World (Oxford Univ. Press 2020), https://doi.org/10.1093/oso/9780190088583.001.0001.

68 David Matthews, *EU Urged to Consider DNA Order Screening to Prevent an Engineered Pandemic*, Science|Business (Jan. 16, 2025), https://sciencebusiness.net/news/biotech/eu-urged-consider-dna-order-screening-prevent-engineered-pandemic [accessed 2 March 2026].

69 Proposal for a Regulation of the European Parliament and of the Council on establishing a framework of measures for strengthening Union’s biotechnology and biomanufacturing sectors particularly in the area of health and amending Regulations (EC) No 178/2002, (EC) No 1394/2007, (EU) No 536/2014, (EU) 2019/6, (EU) 2024/795 and (EU) 2024/1938 (European Biotech Act), COM (2025) 1022 final (Dec. 16, 2025), Articles 42–53, https://health.ec.europa.eu/document/download/ec1475b7-e3f9-409e-b927-fc7e69306a8c_en?filename=biotech_reg-com2025-1022_act_en.pdf. [accessed 2 March 2026].

70 Jasper Grandia & John Meehan, *Public Procurement as a Policy Tool: Using Procurement to Reach Desired Outcomes in Society*, 30 Int'l J. Pub. Sector Mgmt. 302 (2017).

71 Jonathan B. Tucker, *Double-Edged DNA: Preventing the Misuse of Gene Synthesis*, Issues Sci. Tech. 26 (Spring 2010), https://issu.es.org/tucker-2/. [accessed on 2 March 2026].

72 World Health Organization, *supra* note 11, at 49.

73 E.g. Restatement (Third) of Torts: Liability for Physical and Emotional Harm

74 Steven Shavell, *A Model of the Optimal Use of Liability and Safety Regulation*, 15 RAND J. Econ. 271 (1984); Steven Shavell, *Liability for Harm Versus Regulation of Safety*, 13 J. Legal Stud. 357 (1984).

75 U.S. Food & Drug Admin., *Guidances: Drugs* (About FDA Guidances), FDA, https://www.fda.gov/drugs/guidance-compliance-regulatory-information/guidances-drugs; [accessed 2 March 2026]. Paul Craig, Administrative Law (9^th^ ed. Sweet & Maxwell 2021).

76 Organisation for Economic Co-operation and Development, *Managing Risks and Incentivising Growth: Draft Best Practice Principles for Licensing and Permitting* (2025), https://www.oecd.org/en/publications/best-practice-principles-for-licensing-and-permitting_5f63586d-en/full-report.html; *Authority Tools: Pervasive, Persistent and Powerful*, in Routledge Handbook of Policy Design 243 (Michael Howlett & Ishani Mukherjee eds., Routledge 2018).

77 Leach, *supra* note 8.

78 Wheeler et al., *supra* note 22, 71–78.

79
A.L. Rooney & P.R. Van Ostenberg, Licensure, Accreditation, and Certification: Approaches to Health Services Quality (Quality Assurance Project, Center for Human Servs. 1999).

80 Colin Scott, *Standard-Setting in Regulatory Regimes*, in The Oxford Handbook of Regulation 104 (Robert Baldwin, Martin Cave & Martin Lodge eds., 2010); Cary Coglianese & Evan Mendelson, *Meta-Regulation and Self-Regulation*, in The Oxford Handbook of Regulation 146 (Robert Baldwin, Martin Cave & Martin Lodge eds., 2010).

81 Cassese et al., eds., *supra* note 45; Tim Büthe & Walter Mattli, The New Global Rulers: The Privatization of Regulation in the World Economy (Princeton Univ. Press 2011); Scott, *supra* note 80; and Coglianese & Mendelson, *supra* note 80.

82 Carter et al., *supra* note 17; The National Academies of Sciences, *supra* note 19; Aurelia Attal-Juncqua et al., *Biosecurity Governance Across Uncertain Artificial Intelligence Futures: Perspectives from a Side Event on Biosecurity and Frontier AI at the 2025 AI Action Summit*, RAND Corp., CF-A4186-1 (2025), https://doi.org/10.7249/CFA4186-1 [accessed 2 March 2026].

83 International Court of Justice, *Legality of the Threat or Use of Nuclear Weapons*, Advisory Opinion, (8 July 1996, ICJ Reports), 226. https://www.icj-cij.org/sites/default/files/case-related/95/095-19960708-ADV-01-00-EN.pdf; [accessed 2 March 2026]. Dapo Akande et al., *Drawing the Cyber Baseline: The Applicability of Existing International Law to the Governance of Information and Communication Technologies*, 99 Int'l L. Stud. 7 (2021), https://digital-commons.usnwc.edu/ils/vol99/iss1/2/.

84 Tessa Alexanian et al., *The Biological Weapons Convention in the Age of Synthetic Biology*, in 50 Years of the Biological Weapons Convention (Barry de Vries, Kristoffer Burck & Sannimari Veini eds., T.M.C. Asser Press/Springer 2026) *Global Health Security Index: Building Collective Action and Accountability* (2021), https://ghsindex.org/wp-content/uploads/2021/12/2021_GHSindexFullReport_Final.pdf; [accessed 2 March 2026]. Additionally, biosecurity is a key element of the ‘prevention’ pillar in the Global Health Security Index (GHSI).

85 Article 2: ‘The purpose and scope of these Regulations are to prevent, protect against, control and provide a public health response to the international spread of disease …’.

86 Definition of ‘disease’, and Article 2; IHR: World Health Organization, *International Health Regulations (2005)*, Seventy-Seventh World Health Assembly, A77/A/CONF./14 (June 1, 2024), https://apps.who.int/gb/ebwha/pdf_files/WHA77/A77_ACONF14-en.pdf. [accessed 2 March 2026].

87 World Health Organization, *supra* note 86.

88 World Health Organization, *Joint External Evaluation Tool: International Health Regulations (2005)*, Third Edition, WHO, (2022), https://iris.who.int/bitstream/handle/10665/357087/9789240051980-eng.pdf?sequence=1. [accessed 2 March 2026].

89 World Health Organization, *supra* note 88, at 44–50.

90 World Health Organization, Intergovernmental Negotiating Body, https://inb.who.int/ [accessed 2 March 2026].

91 Article 12(5)(e) also now acknowledges that the Pathogen Access and Benefit Sharing System (PABS) thereunder shall be consistent with other biosecurity frameworks: ‘(e) implementation consistent with applicable international law and with applicable national and domestic law, regulations and standards related to risk assessment, biosafety, biosecurity and export control of pathogens, and data protection’.

92 United Nations Office for Disarmament Affairs, *Membership and Regional Groups*, https://disarmament.unoda.org/biological-weapons/about/membership-and-regional-groups/ [accessed 2 March 2026].

93
*Additional Understandings and Agreements Reached by Previous Review Conferences Relating to Each Article of the Convention*, BWC/CONF.IX/PC/5 (Jan. 10, 2022), ¶ 7.

94 Akande et al., *supra* note 83; Alexanian et al., *supra* note 84.

95 Braden Leach, *Necessary Measures: Synthetic Biology & the Biological Weapons Convention*, 25 Stan. Tech. L. Rev. 141 (2021), https://law.stanford.edu/publications/necessary-measures-synthetic-biology-the-biological-weapons-convention/; Isaac Heron, *Navigating the Risks of Artificial DNA: Do We Need an International Treaty?*, UOtaLawTD 13, University of Otago Legal Studies Research Paper No. 13/2023 (2023), at 32, https://www.nzlii.org/nz/journals/UOtaLawTD/2023/13.html [accessed 2 March 2026].

96 Sally Longworth, *Interpreting the Biological Weapons Convention—What Are ‘Necessary Measures’ under Article IV of the Convention?*, FOI Memo No. 8407 (Swedish Defence Research Agency, Dec. 27, 2023), https://www.foi.se/rest-api/report/FOI%20Memo%208407#:~:text=What%20is%20%E2%80%9Cnecessary%E2%80%9D%20must%20be,BWC%20and%20anything%20in%20between. [accessed 2 march 2026].

97 Heron, *supra* note 95, at 42; Alexanian et al., *supra* note 84.

98 Heron, *supra* note 95, at 43; Alexanian et al., *supra* note 84.​

99 BWC/WG/6/CRP.1, Draft Specific and Effective Measures, Including Possible Legally-Binding Measures, to Strengthen and Institutionalize the Biological Weapons Convention in All Its Aspects: Rolling Text by the Chairperson of the Working Group on the Strengthening of the Convention, Working Group on the Strengthening of the Biological Weapons Convention, Sixth Session, Geneva, Aug. 11–22, 2025 (Aug. 7, 2025). https://docs-library.unoda.org/Biological_Weapons_Convention_-Working_Group_on_the_strengthening_of_the_ConventionSixth_session_(2025)/BWC-WG-6-1-CRP1_draft_rolling_text.pdf. [accessed 2 March 2026].

100 Alexanian et al., *supra* note 84.

101 Australia Group, *Introduction*, https://www.dfat.gov.au/publications/minisite/theaustraliagroupnet/site/en/introduction.html [accessed 2 March 2026].

102 Singapore Customs, *Frequently Asked Questions on Updates to the Strategic Goods (Control) Order, Strategic Goods (Control) (Brokering) Order, and Strategic Goods (Control) Regulations*, https://www.customs.gov.sg/files/businesses/tssb/frequently%20asked%20questions_sgco2023.pdf. [accessed 2 March 2026].

103 Australia Group, *Common Control Lists*, https://www.dfat.gov.au/publications/minisite/theaustraliagroupnet/site/en/common-control-lists.html [accessed 2 March 2026].

104 Australia Group, *List of Human and Animal Pathogens and Toxins for Export Control*, https://www.dfat.gov.au/publications/minisite/theaustraliagroupnet/site/en/human_animal_pathogens.html [accessed 2 March 2026].

105 It defines genetic elements as follows: ‘Genetic elements include, inter alia: chromosomes, genomes, plasmids, transposons, vectors, and inactivated organisms containing recoverable nucleic acid fragments, whether genetically modified or unmodified, or chemically synthesized in whole or in part’.

106 Australia Group, *List of Human and Animal Pathogens and Toxins for Export Control*, https://www.dfat.gov.au/publications/minisite/theaustraliagroupnet/site/en/human_animal_pathogens.html [accessed 2 March 2026]. The list expressly applies to genetic sequences, stating that: **Genetic Elements and Genetically-modified Organisms**: ‘Any genetically-modified organism which contains, or genetic element that codes for, any of the following: any gene or genes, translated product, or translated products, specific to any listed virus; or any gene or genes specific to any listed bacterium or fungus, and which in itself or through its transcribed or translated products represents a significant hazard to human, animal or plant health, or could endow or enhance pathogenicity; or any listed toxins or their sub-units’.

107 Global Health Security Index, *supra* note 84.

108
*Id*.

109
*Id.*; Mely Caballero-Anthony et al., *Emerging Biosecurity Landscape in Southeast Asia-Updated Report (Executive Summary)*, Centre for Non-Traditional Security Studies, S. Rajaratnam School of International Studies, (Mar. 17, 2025), https://www.rsis.edu.sg/wp-content/uploads/2025/03/Emerging-Biosecurity-Landscape-in-Southeast-Asia_Executive_Summary.pdf. [accessed 2 March 2026].

110 Global Health Security Index, *supra* note 84, at 57, at 9–10.

111 Global Health Security Index, *supra* note 84, at p. 57.

112 National Health Security Act 2007 (Cth), Department of Health, *Security Sensitive Biological Agents Regulatory Scheme*—*Fact Sheet 5: List of Security Sensitive Biological Agents* 3 (2016) (Austl.)

113 Infectious Disease Control and Prevention Act 2009 (Korea), Arts 2(19) and 21(1).

114 Health of Animals Act, S.C. 1990, c. 21 (Can.); International Biosecurity and Biosafety Initiative for Science, *Policy Spotlight: French Leadership in Defining and Regulating Genetic Fragments*, https://ibbis.bio/policy-spotlight-french-leadership-defining-and-regulating-genetic-fragments/ [accessed 2 March 2026].

115
*Biosecurity Law of the People's Republic of China*, Chapter I: General Provisions (law effective Apr. 15, 2021), https://faolex.fao.org/docs/pdf/chn198696E.pdf [accessed 2 March 2026]. I have not found any publications on this issue, but I have been informed by a legal Chinese scholar working in this field that ‘there is no established approach specifically for regulating SNA in China. China has not yet issued SNA screening guidelines similar to those published in the US and UK.’

116 UK Screening Guidance on Synthetic Nucleic Acids for Users and Providers (Gov-uk 2024), https://www.gov.uk/government/publications/uk-screening-guidance-on-synthetic-nucleic-acids/uk-screening-guidance-on-synthetic-nucleic-acids-for-users-and-providers [accessed 2 March 2026].

117 Ayelet Berman et al., *Introduction: Rethinking Stakeholder Participation in Global Governance*, in Rethinking Participation in Global Governance: Voice and Influence After Stakeholder Reforms in Global Finance and Health (Joost Pauwelyn et al. eds., Oxford Univ. Press 2022); N. Jansen Calamita & Ayelet Berman (eds.), Investment Treaties and the Rule of Law (Cambridge Univ. Press 2017); Caballero-Anthony et al., *supra* note 109.

118 cbd-ts-100-en.pdf

119 Global Health Security Index, *supra* note 84; see 2021 GHS Index Country Profile for [country] country score justification summary, eg, https://ghsindex.org/country/singapore/.

120 IBBIS, *supra* note 114.

121 2021 GHS Index Country Profile for United States, https://ghsindex.org/country/united-states/.

122 UK screening guidance on synthetic nucleic acids for users and providers, *supra* note 116.

123 See the overview of the legislative framework at UK Screening Guidance on Synthetic Nucleic Acids for Users and Providers, *supra* note 116. ‘any nucleic acid deriving from a micro-organism listed in this schedule (synthetic or naturally derived, contiguous or fragmented …) that can encode infectious or replication competent forms of any of the listed microorganisms’, ‘any nucleic acid sequence derived from the micro-organism which when inserted … alters or enhances that organism’s ability to cause serious harm to human health’.

124 UK Biological Security Strategy (Gov-uk 2024), https://www.gov.uk/government/publications/uk-biological-security-strategy/uk-biological-security-strategy-html [accessed 2 March 2026].

125 UK Screening Guidance on Synthetic Nucleic Acids for Users and Providers, *supra* note 116.

126 Draft Report on the Future of the EU Biotechnology and Biomanufacturing Sector: Leveraging Research, Boosting Innovation and Enhancing Competitiveness, EUR. PARL. DOC. 2025/2008(INI) (2025), at 5.

127 Communication from the Commission to the European Parliament, the Council, the European Economic and Social Committee and the Committee of the Regions, Building the Future with Nature: Boosting Biotechnology and Biomanufacturing in the EU, COM (2023) 800 final (Dec. 20, 2023), at 17–18.

128 Public Health Security and Bioterrorism Preparedness and Response Act of 2002, Pub. L. No. 107–188, 116 Stat. 594 (2002).

129
*Select Agents and Toxins List*, Fed. Select Agent Program (accessed 2 March 2026), https://www.selectagents.gov/sat/list.htm.

130
*Id*.

131 7 C.F.R. § 331; 9 C.F.R. § 121; 42 C.F.R. § 73 (regulating HHS and USDA Select Agents and Toxins); 42 C.F.R. § 73.3(c) Genetic Elements, Recombinant and/or Synthetic Nucleic Acids, and Recombinant and/or Synthetic Organisms: (1) Nucleic acids that can produce infectious forms of any of the select agent viruses listed in paragraph (b) of this section.

132 42 C.F.R. § 73.3(c)(1) (2024).

133 42 C.F.R. § 73.3(c)(2).

134
*Guidance on the Regulation of Select Agent and Toxin Nucleic Acids*, Fed. Select Agent Program (Feb. 2020), https://www.selectagents.gov/compliance/guidance/nucleic/index.htm. [accessed 2 March 2026].

135 Leach, *supra* note 8.

136 42 C.F.R. § 73.7(a).

137 42 C.F.R. §§ 73.7(a), 73.16(a) and (b).

138 15 C.F.R. § 738.

139 15 C.F.R. § 774, supp. 1, ECCN 1C353.

140 Technical Notes (2), 15 C.F.R. § 774, supp. 1, ECCN 1C353.

141 22 CFR § 121.1—The United States Munitions List.

142 National Science Advisory Board for Biosecurity, *supra* note 57.

143 U.S. Dep’t of Health & Human Servs., *Screening Framework Guidance for Providers of Synthetic Double-Stranded DNA* (2010), https://obamawhitehouse.archives.gov/sites/default/files/omb/assets/omb/inforeg/screening_framework_guidance.pdf. [accessed 2 March 2026].

144 Curtis Matthew Sharkey et al., *Enhancing Gene Synthesis Security: An Updated Framework for SNA Screening and the Responsible Use of Synthetic Biological Materials*, 29 Applied Biosafety 63 (2024), https://doi.org/10.1089/apb.2023.0036.

145 U.S. Dep’t of Health & Human Servs., *Screening Framework Guidance for Providers and Users of Synthetic Nucleic Acids* (2023), https://aspr.hhs.gov/legal/syndna/Documents/Synthetic-Nucleic-Acid-Screening-Framework.pdf.

146 U.S. Department of Health & Human Services, *supra* note 145, at 8.

147 U.S. Department of Health & Human Services, *supra* note 145, at 2.

148 U.S. Department of Health & Human Services, *supra* note 145, at 6–7.

149 U.S. Department of Health & Human Services, *supra* note 145, at 6.

150 Kevin M. Esvelt, *It Shouldn’t Be Easy to Buy Synthetic DNA Fragments to Recreate the 1918 Flu Virus*, STAT (May 8, 2024), https://www.statnews.com/2024/05/08/shouldnt-be-easy-buy-synthetic-dna-fragments-recreate-deadly-1918-flu-virus/. [accessed 2 March 2026].

151 Article 4.4, Exec. Order No. 14110, *supra* note 40.

152
*Framework for Nucleic Acid Synthesis Screening*, Exec. Office of the President, Office of Science & Technology Policy (Apr. 29, 2024), https://bidenwhitehouse.archives.gov/ostp/news-updates/2024/04/29/framework-for-nucleic-acid-synthesis-screening/; *Framework for Nucleic Acid Synthesis Screening*, National Science & Technology Council, Fast Track Action Communication on SNAProcurement Screening (Apr. 2024), https://bidenwhitehouse.archives.gov/wp-content/uploads/2024/04/Nucleic-Acid_Synthesis_Screening_Framework.pdf. [accessed 2 March 2026].

153 National Science & Technology Council, *Framework for Nucleic Acid Synthesis Screening* (Sept. 2024), https://www.whitehouse.gov/wp-content/uploads/2024/09/Nucleic-Acid-Synthesis-Screening-Framework.pdf.

154 Emily Mullin, *The U.S. Is Cracking Down on Synthetic DNA*, WIRED (May 6, 2024), https://www.wired.com/story/synthetic-dna-us-biden-regulation/ [accessed 2 March 2026].

155
*Executive Order 14292: Improving the Safety and Security of Biological Research*, Exec. Order No. 14,292, 90 Fed. Reg. ___ (May 8, 2025), https://www.govinfo.gov/content/pkg/DCPD-202500561/pdf/DCPD-202500561.pdf

156 Assemb. B. 70, 2021–2022 Leg., Reg. Sess. (Cal. 2021).

157 S. 2400, 118th Cong. (2023).

158 European Commission, *supra* note 69.

159 Gene Technology Bill (N.Z.), Bill No. 110–1 (2024).

160 Assemb. B. 70, *supra* note 156.

161 S. 2400, *supra* note 157.

162 Gene Technology Bill (N.Z.), *supra* note 159 at cl. 157.

163 Gene Technology Bill (N.Z.), *supra* note 159 at), cls. 65–69.

164 European Commission, *supra* note 69.

165 International Gene Synthesis Consortium, *IGSC Launch Announcement* (2009), https://genesynthesisconsortium.org/wp-content/uploads/IGSC-Launch-Announcement.pdf. [accessed on 2 March 2026].

166 International Gene Synthesis Consortium, *supra* note 58.

167 International Gene Synthesis Consortium, *Members*, https://genesynthesisconsortium.org/ (accessed 2 March 2026).

168 International Gene Synthesis Consortium, *Harmonized Screening Protocol v2.0*, 1 (2017), https://genesynthesisconsortium.org/wp-content/uploads/IGSCHarmonizedProtocol11-21-17.pdf. [accessed 2 March 2026].

169 Int’l Gene Synthesis Consortium, *IGSC Membership Application* (Nov. 21, 2017), https://genesynthesisconsortium.org/wp-content/uploads/IGSCMembershipApplication11-21-17.pdf [accessed 2 March 2026].

170 International Gene Synthesis Consortium, *IGSC Harmonized Screening Protocol* (Version 3.0, Jan. 2022), https://genesynthesisconsortium.org/wp-content/uploads/IGSC-Harmonized-Screening-Protocol-v3.0-1.pdf [accessed 2 March 2026].

171
*Id.*

172
*International Gene Synthesis Consortium, supra note 170 at* Requirement 7.2.2 at 16, https://genesynthesisconsortium.org/wp-content/uploads/IGSC-Harmonized-Screening-Protocol-v3.0-1.pdf.

173
*Id*.

174 International Gene Synthesis Consortium, *supra* note 170, Requirements 7.1.3 & 7.1.5, at 15 (2024), https://genesynthesisconsortium.org/wp-content/uploads/IGSC-Harmonized-Screening-Protocol-v3.0-1.pdf.

175 International Gene Synthesis Consortium, *supra* note 170, Requirement 7.1.2, at 15 (Sept. 3, 2024), https://genesynthesisconsortium.org/wp-content/uploads/IGSC-Harmonized-Screening-Protocol-v3.0-1.pdf.

176 GenScript USA Incorporated, *Framework for Nucleic Acid Synthesis Screening: Formal Statement from GenScript* (Oct. 8, 2024), https://www.genscript.com/gsfiles/techfiles/Framework_for_Nucleic_Acid_Synthesis_Screening_Formal_Statement_from_GenScript.pdf. [accessed 2 March 2026].

177 Maxmen Aida, *Why a Misleading Red-Team Study of the Gene Synthesis Industry Wrongly Casts Doubt on Industry Safety, Bulletin* (June 2024), https://thebulletin.org/2024/06/why-a-misleading-red-team-study-of-the-gene-synthesis-industry-wrongly-casts-doubt-on-industry-safety/. [accessed 2 March 2026].

178 International Organization for Standardization, *About ISO*, https://www.iso.org/about [accessed 2 March 2026].

179 International Organization for Standardization, *ISO 20688-2:2024, Biotechnology—Nucleic Acid Synthesis—Part 2: Requirements for the Production and Quality Control of Synthesized Gene Fragments, Genes, and Genomes*, Part 1 (2024), https://www.iso.org/standard/75852.html. [accessed 2 March 2026].

180
*Id*.

181 International Organization for Standardization, *supra* note 179, Part 4.1–4.4.

182 International Organization for Standardization, *supra* note 179, Part 6.1–6.2.

183 International Organization for Standardization, *supra* note 179, Part 4.4.

184 International Organization for Standardization, *supra* note 179, Part 6.2.

185 International Organization for Standardization, *supra* note 179, Part 3.11.

186 International Organization for Standardization, *supra* note 179, Part 4.4.

187 Responsible AI x Biodesign, *supra* note 42; Callaway, *supra* note 42.

188 iGEM, *supra* note 43.

189 WHO, *supra* note 11.

